# On the average temperature of airless spherical bodies and the magnitude of Earth’s atmospheric thermal effect

**DOI:** 10.1186/2193-1801-3-723

**Published:** 2014-12-10

**Authors:** Den Volokin, Lark ReLlez

**Affiliations:** Tso Consulting, 843 E Three Fountains Suite 260, Salt Lake City, UT 84107 USA

**Keywords:** Planetary temperature, Analytic model, Atmospheric thermal enhancement, Greenhouse effect, Thermodynamic enhancement, Hölder’s inequality

## Abstract

The presence of atmosphere can appreciably warm a planet’s surface above the temperature of an airless environment. Known as a natural Greenhouse Effect (GE), this near-surface Atmospheric Thermal Enhancement (ATE) as named herein is presently entirely attributed to the absorption of up-welling long-wave radiation by greenhouse gases. Often quoted as 33 K for Earth, GE is estimated as a difference between planet’s observed mean surface temperature and an effective radiating temperature calculated from the globally averaged absorbed solar flux using the Stefan-Boltzmann (SB) radiation law. This approach equates a planet’s average temperature in the absence of greenhouse gases or atmosphere to an effective emission temperature assuming ATE ≡ GE. The SB law is also routinely employed to estimating the mean temperatures of airless bodies. We demonstrate that this formula as applied to spherical objects is mathematically incorrect owing to Hölder’s inequality between integrals and leads to biased results such as a significant underestimation of Earth’s ATE. We derive a new expression for the mean physical temperature of airless bodies based on an analytic integration of the SB law over a sphere that accounts for effects of regolith heat storage and cosmic background radiation on nighttime temperatures. Upon verifying our model against Moon surface temperature data provided by the NASA Diviner Lunar Radiometer Experiment, we propose it as a new analytic standard for evaluating the thermal environment of airless bodies. Physical evidence is presented that Earth’s ATE should be assessed against the temperature of an equivalent airless body such as the Moon rather than a hypothetical atmosphere devoid of greenhouse gases. Employing the new temperature formula we show that Earth’s *total* ATE is ~90 K, not 33 K, and that ATE = GE + TE, where GE is the thermal effect of greenhouse gases, while TE > 15 K is a thermodynamic enhancement independent of the atmospheric infrared back radiation. It is concluded that the contribution of greenhouse gases to Earth’s ATE defined as GE = ATE – TE might be greater than 33 K, but will remain uncertain until the strength of the hereto identified TE is fully quantified by future research.

**Electronic supplementary material:**

The online version of this article (doi:10.1186/2193-1801-3-723) contains supplementary material, which is available to authorized users.

## Background

It is an undisputed fact that the atmosphere can appreciably heat a planet’s surface above the temperature of an airless environment receiving the same stellar irradiance. Known as a natural Greenhouse Effect (GE), this extra atmospheric warmth is presently completely attributed to the absorption and re-emission of upwelling long-wave radiation by heat-absorbing gases such as CO_2_, water vapor, methane (CH_4_), nitrous oxide (N_2_O) and others (Schmidt et al. 2010; Lacis et al. 2010). Thus, GE has two scientific measures at the present (Lacis et al. 2013): *a*) as an observed difference in the outgoing global infrared flux (W m^−2^) between the planet surface and the top of the atmosphere (Ramanathan and Inamdar 2006; Schmidt et al. 2010; Pierrehumbert 2011); and *b*) as an extra warmth or increased temperature at the surface (Hansen et al. 1981; Schmidt et al. 2010; Lacis et al. 2010, 2013). This study explores the latter measure of GE using Earth as an example. The additional warmth provided by GE creates climate conditions that foster life on our Planet by enabling the existence of liquid oceans and providing for a global water cycle (Pierrehumbert 2010). In order to better distinguish between the two measures of GE and to facilitate a proper understanding of our analysis and results, we hereto introduce the term *Atmospheric Thermal Enhancement* (ATE) to describe the *total* extra warmth near a planet surface measured as a difference (K) between the planet’s present mean global surface temperature and an estimated planetary reference temperature in the *absence* of atmosphere. By referring to the whole atmosphere, ATE also allows for investigation of potential contributions beyond those currently attributed to greenhouse gases.

According to satellite observations, Earth’s atmosphere retains on average 155–158 W m^−2^ of the upwelling long-wave radiation emitted by the surface (Kiehl and Trenberth 1997; Trenberth et al. 2009; Stephens et al. 2012; Wild et al. 2013). This infrared heat absorption by greenhouse gases a.k.a. long-wave radiative forcing (Kiehl and Trenberth 1997) is presently believed to drive 100% of the near-surface ATE (Peixoto and Oort 1992; Lacis et al. 2010; Pierrehumbert 2010; Schmidt et al. 2010). Most researchers assume that greenhouse gases boost the Earth’s mean global surface temperature by about 33 K (e.g. Hansen et al. 1981; Peixoto and Oort 1992; Wallace and Hobbs 2006; Lacis et al. 2010, 2013; Schmidt et al. 2010). Some argue that Earth’s GE is only ~20 K (e.g. Zeng 2010). Knowing the exact magnitude of this natural atmospheric effect is important because it might relate to the planet’s long-term climate sensitivity to anthropogenic greenhouse emissions. The goal of this study is to examine the current method for calculating the thermal effect of planetary atmospheres and reassess the magnitude of Earth’s ATE as an example using a new approach to estimating the average global temperature of *airless* celestial bodies validated against recent NASA observations and model simulations of the Moon thermo-physical environment.

### Stefan-Boltzmann radiation law

According to the Stefan-Boltzmann (SB) law, any physical object with a temperature above the absolute zero emits radiation with a total intensity that is proportional to the 4th power of the object’s absolute surface temperature. This implies that an object’s equilibrium surface temperature (*T*, K) can be calculated from the amount of absorbed radiation (*I*, W m^−2^) using the relation

1 $$T={\left(\frac{I}{\varepsilon \sigma }\right)}^{\frac{1}{4}}$$

where *ε* is the object’s broadband thermal emissivity/absorptivity (0 ≤ *ε* ≤ 1) and *σ* = 5.6704 × 10^−8^ W m^−2^ K^−4^ is the SB constant. A theoretical blackbody has *ε* = 1.0, while the emissivity of real objects such as soil and regolith is typically in the range 0.95 ≤ *ε* ≤ 0.99 for far infrared wavelengths. A key assumption of Eq. (1) is that the object has an isothermal surface, which absorbs and emits a spatially homogeneous flux of radiation *I*.

### Current application of the SB law to planetary bodies

The absorption of solar radiation by a spherical body varies with latitude and the time of day as a function of the solar incidence angle and the local surface albedo. However, the globally averaged flux of absorbed solar radiation *S*_*a*_ (W m^−2^) can reliably be calculated using the formula:

2 $$S_{a}=\frac{S_{o}}{4}\left(1-\;\alpha_{p}\right)$$

where *S*_*o*_ is the solar irradiance (W m^−2^), i.e. the flux incident on a plane perpendicular to the solar rays at the top of the atmosphere (TOA), and *α*_*p*_ is the planetary Bond albedo (decimal fraction). The factor 1/4 serves to distribute the solar flux from a flat surface to a sphere and arises from the fact that the surface area of a sphere is 4 times larger than the surface area of a disk with the same radius. Hence, it seems logical that one could calculate a global equilibrium temperature for a planet from *S*_*a*_ using the SB law, i.e.

3 $$T_{e}=\;{\left(\frac{S_{a}}{\varepsilon \;\sigma }\right)}^{1/4}={\left[\frac{S_{o}\;\left(1-\alpha_{p}\right)}{4\;\varepsilon \;\sigma }\right]}^{1/4}$$

In this expression, *T*_e_ is known as ‘*effective-emission*’ or ‘*radiating equilibrium*’ temperature (K), since it corresponds to the globally averaged radiation flux absorbed by a celestial body. Hereafter, we use the term *effective emission temperature* to denote quantities calculated from spatially averaged fluxes of absorbed solar radiation. This is in contrast to other terms we use such as ‘*mean physical*’, ‘*average equilibrium*’*,* or ‘*average skin*’ temperature that refer to quantities obtained via area-weighted averaging of observable or measured surface temperatures.

Equations (2) and (3) were first introduced to planetary science in the early 1960s (Blanco and McCuskey 1961; Möller 1964) and have been utilized ever since to estimate the average global temperatures of airless or nearly airless celestial bodies such as Mercury, Moon and Mars (e.g. Williams 2014), to quantify the strength of greenhouse effects of planetary atmospheres (e.g. Hansen et al. 1981; Lacis et al. 2013), and to determine the boundaries of Habitable Zones around stars (e.g. Kaltenegger and Sasselov 2011; Schulze-Makuch et al. 2011).

Employing typical values for Earth, i.e. *S*_*o*_ = 1,360.9 W m^−2^ (Kopp and Lean 2011), *α*_*p*_ = 0.294 (Loeb et al. 2009; Stephens et al. 2012; Wild et al. 2013) and assuming *ε* = 1.0, formulas (2) and (3) yield *S*_*a*_ = 240.2W m^−2^ and *T*_e_ = 255.1 K, respectively. The latter estimate is the basis for the frequently quoted 255 K (−18 C) mean global temperature of Earth in the absence of GE, i.e. if the Earth’s atmosphere were absent or completely transparent to the outgoing infrared radiation (e.g. Pierrehumbert 2010). According to the NOAA National Climatic Data Center, Earth’s observed mean surface temperature (*T*_s_) has been stable over the past 16 years and equals 287.6 K (+14.47 C). Thus, the current method quantifies GE as *T*_s_ – *T*_e_ = 287.6 – 255.1 = 32.5 K. Most studies assume a planetary albedo of 0.3 and arrive at GE ≈ 33 K. The present Greenhouse theory attributes Earth’s entire atmospheric thermal effect to the absorption and re-emission of outgoing long-wave radiation by tropospheric greenhouse gases assuming ATE ≡ GE (Hansen et al. 1981; Peixoto and Oort 1992; Wallace and Hobbs 2006; Marshall and Plumb 2008; Pierrehumbert 2010; Schmidt et al. 2010; Schulze-Makuch et al. 2011; Lacis et al. 2010, 2013).

Some authors (e.g. Zeng 2010) argue that the 33 K GE estimate rests on a logical caveat, since it is based on a reference temperature computed from Eq. (3) using Earth’s full albedo *α*_*p*_ = 0.3 that includes the radiative effects of clouds and water vapor. In order for a temperature to be able to serve as a proper reference in this case it must describe the planet’s surface thermal environment in the *absence* of greenhouse gases. Removing heat-absorbing gases from Earth’s atmosphere, of which water vapor is primary (Schmidt et al. 2010; Lacis et al. 2013), would reduce the Earth albedo well below 0.294, since the scattering of sunlight by clouds and airborne water molecules accounts for about 50% of the planet’s total shortwave reflectance. Hence, quantifying the strength of GE logically requires using a surface albedo (*α*_*o*_) in Eq. (3) that is free from the radiative effects of atmospheric water (Zeng 2010). Following a similar logic, we argue that Earth’s *total* ATE ought to be evaluated against the temperature of an equivalent *airless* body rather than a hypothetical atmosphere devoid of greenhouse gases. This is because, in addition to vapor clouds, air molecules and airborne aerosols significantly contribute to the atmospheric albedo as well as for other reasons related to the planet’s surface thermal conductivity explained below.

Recent analyses of Earth’s global energy budget based on satellite observations (Stephens et al. 2012) and ground measurements (Wild et al. 2013) suggest 0.122 ≤ *α*_*o*_ ≤ 0.13 for the Earth averaged land-ocean albedo. Serendipitously, these values are similar to the Moon’s 0.136 average broadband albedo measured by the Clouds and the Earth’s Radiant Energy System (CERES) (Matthews 2008) and the 0.131 effective lunar-regolith albedo estimated in this study (see discussion below). Using the satellite-observed value *α*_*o*_ = 0.122 in Eq. (3) produces *T*_e_ = 269.4 K for Earth, which translates into ATE ≡ GE = 287.6 – 269.4 = 18.2 K according to the present method based on the effective emission temperature. Zeng (2010) arrived at ATE ≈ 20 K by assuming a somewhat higher Earth surface albedo *α*_*o*_ = 0.14. We concur that, in the context of Eq. (3), the 18–20 K estimate of ATE is theoretically more justifiable than the canonic 33 K value obtained by employing Earth’s total albedo. It is important to note that all popular estimates of the atmospheric thermal effect ranging from 18 K to 33 K are based on Eq. (3) or similar 1-D radiative-transfer models and were not derived from 3-D global circulation models.

The above discussion makes it clear that quantifying the *total* magnitude of ATE requires an accurate estimation of the planet’s equilibrium mean surface temperature in the *absence* of atmosphere. In general, we hereto refer to such a ‘*no-atmosphere*’ estimate as the average skin temperature (*T*_na_) of an Airless Spherical Celestial Object (ASCO). Obviously, *T*_na_ depends on solar irradiance and the surface albedo, and ATE = *T*_s_ – *T*_na_. Current climate and planetary sciences oftentimes identify mean physical temperatures of airless celestial bodies with their effective emission temperatures implicitly assuming *T*_s_ ≡ *T*_na_ (e.g. Schulze-Makuch et al. 2011; Lacis et al. 2013). For example, the average global temperatures reported by the NASA Planetary Factsheet (Williams 2014) for the Moon (270.7 K), Mercury (440 K) and even Mars (210 K) have been calculated from Eq. (3). However, there is a theoretical problem with this formula as applied to spherical bodies related to what is known in mathematics as Hölder’s inequality between integrals (Beckenbach and Bellman 1983; Abualrub and Sulaiman 2009). The problem has been identified by previous research (e.g. Leconte et al. 2013), but it has not been thoroughly analyzed in terms of its implications for the physical meaning and usefulness of *T*_e_.

### Hölder’s inequality and its implications for planetary flux-temperature relationships

In its general form, Hölder’s inequality states that, for any pair of measurable real- or complex-valued functions *f*(*x*) and *g*(*x*), the following relationship is always true

4a $$\int \left|f\left(x\right)\;g\left(x\right)\right|\;dx\;\leq \;{\left\{\int {\left|f\left(x\right)\right|}^{p}dx\right\}}^{\frac{1}{p}\;}{\left\{\int {\left|g\left(x\right)\right|}^{q}dx\right\}}^{\frac{1}{q}}$$

provided 1 ≤ *p*, *q* < ∞ and 1/*p* + 1/*q* = 1 (Beckenbach and Bellman 1983). In regard to the SB law and the latitudinal distribution of equilibrium temperatures *T*(*μ*) on the surface of a sphere (where 0 ≤ *μ* ≤ 1 is an area-weighting factor defined as the cosine of latitude), the relevant form of Hölder’s inequality is obtained from (4a) by substituting *f*(*x*) = *T*(*μ*), *g*(*x*) = 1, *p* = 4 and *q* = 4/3. This produces:

4b $$\int_{0}^{1}T\left(\mu \right)\;d\mu \;<\;{\left\{\overset{1}{\underset{0}{\int }}T{\left(\mu \right)}^{4}\;d\mu \right\}}^{\frac{1}{4}}$$

Inequality (4b) implies that the area-weighted average temperature of a spherical surface (on the left) is always *lower* than the temperature calculated from the area-weighted average long-wave radiation emitted by the surface in proportion to *T*(*μ*)^4^ (on the right). Due to a non-linear relationship between temperature and the emitted radiative bolometric flux, and a strong latitudinal variation of the absorbed shortwave radiation across the surface of a sphere, the actual mean global temperature of a directionally illuminated planet is *not* estimable in principle from a planetary averaged radiative flux (Eq. 2) using the SB law (Eq. 1). This is because a spherical geometry violates the fundamental assumption in the SB relationship for spatial homogeneity of radiation absorption and emission. Hence, Eq. (3) yields the temperature of a flat isothermal surface rather than the average temperature of a thermally heterogeneous sphere as required for planets. In other words, *T*_e_ is the equilibrium temperature of a *black disk* orthogonally illuminated by shortwave radiation with intensity equal to the average solar flux absorbed by a sphere having a Bond albedo *α*_*p*_. This makes *T*_e_ a *non-physical* temperature with respect to a spherical surface. The effect of Hölder’s inequality can be illustrated with the following example.

**Figure 1:**
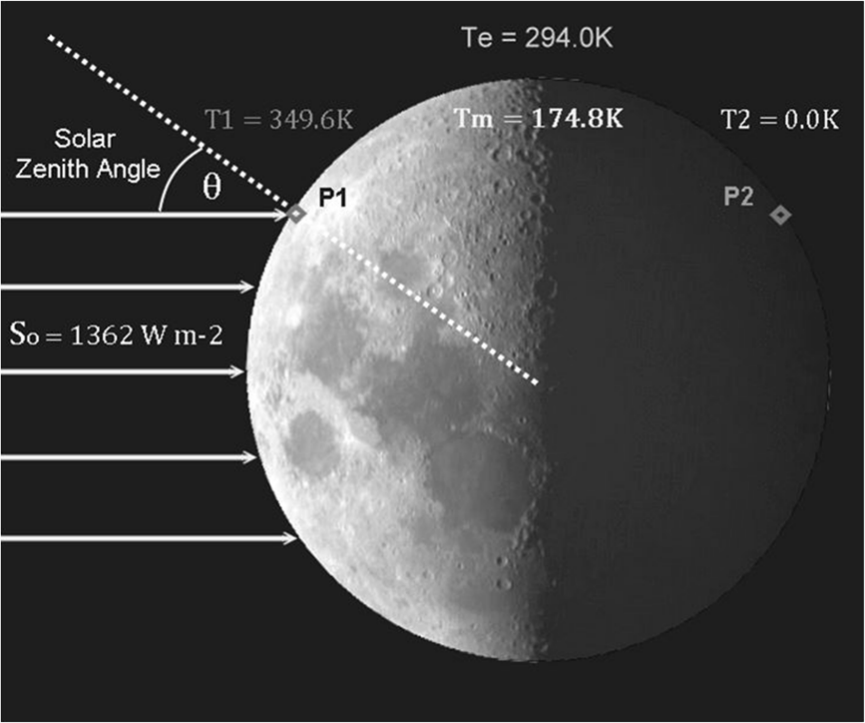
**Illustration of Hölder’s inequality between integrals.** Due to a nonlinearity of the SB law and a non-uniform distribution of the incident solar radiation on the surface of a sphere, the equilibrium temperature (*T*_e_) computed from a spatially averaged radiation flux is always higher than the arithmetic average temperature (*T*_m_).

Consider two points, P_1_ and P_2_, on the surface of an ASCO located at the exact same latitude (e.g. 45°N) but at opposite longitudes so that, when P_1_ is fully illuminated, P_2_ is completely shaded and vice versa (Figure 1). If such an ASCO orbited the Sun at Earth’s distance, had a regolith of zero thermal conductivity, and were only heated by solar radiation, then the equilibrium temperature of the illuminated point would be *T*_1_ = [*S*_*o*_ (1 – *α*_*o*_)cos*θ*/*εσ*]^0.25^ = 349.6 K assuming *α*_*o*_ = 0.12 (a typical value for rocky surfaces), a solar incident angle *θ* = 45°, and *ε* = 1.0. The temperature of the shaded point would be *T*_2_ = 0, because it receives no radiation since cos *θ* < 0 and there is no heat release from the regolith at night due to zero heat storage. The mean physical temperature between the two points is simply then *T*_m_ = (*T*_1_ = *T*_2_)/2 = 174.8 K. However, if one employs the average solar flux absorbed between the two points, i.e. *S*_m_ = {[*S*_*o*_ (1 − *α*_*o*_)cos *θ*] + 0}/2 = 423.4 W m^−2^ to calculate a ‘mean’ effective emission temperature, one obtains *T*_e_ = [*S*_m_/*εσ*]^0.25^ = 294.0 K. Clearly *T*_e_ ≫ *T*_m_, a result of Hölder’s inequality.

The conclusion from the above discussion is that a proper calculation of the mean *physical* temperature of an airless celestial body (*T*_na_) requires an explicit integration of the SB law over the planet surface. This means *first* taking the 4th root of the absorbed shortwave flux at every point on the planet and *then* averaging the resulting temperature field across the entire surface rather than calculating a single temperature from the globally averaged absorbed solar flux as done in Eq. (3). It should be pointed out that global climate models intrinsically account for Hölder’s inequality by virtue of being three-dimensional and explicitly resolving the spatial heterogeneity of radiation absorption and emission (as well as other energy transport processes) within the context of a spherical geometry. However, 3-D models have not historically been applied to assess the strength of Earth’s ATE (GE). Hence, our critique is strictly directed towards the effective emission-temperature formula (3) and other similar 1-D radiative-transfer models (e.g. Manabe and Möller 1961; Manabe and Strickler 1964).

From the standpoint of Hölder’s inequality, one would expect *T*_na_ to approach *T*_e_ only if the absorbed solar radiation were uniformly distributed throughout the entire planet surface. However, this requires a regolith of *infinite* lateral thermal conductivity, which is physically impossible. Real ASCOs such as the Moon have extremely low surface thermal conductivities (Vasavada et al. 2012), and the absorbed solar flux varies greatly with latitude and solar angle resulting in a highly non-uniform distribution of surface temperatures. Hence, in the general case, we expect *T*_na_ ≪ *T*_e_. This implies that effective emission temperatures are not equivalent to and should not be confused with actual physical temperatures on a sphere, a conclusion also reached by Leconte et al. (2013). Some researchers identify Earth’s *T*_e_ ≈ 255 K with the observed average temperature at about 5 km altitude in the free troposphere (e.g. Hansen et al. 1981; Marshall and Plumb 2008; Pierrehumbert 2010). Others relate *T*_e_ of airless bodies to brightness temperatures retrieved via radio waves for ~1 m depth below the surface (e.g. Lissauer and Pater 2013, Chapter 4.1). However, Hölder’s inequality reveals that the effective emission temperature of a spherical object is a mathematical abstraction with no physical analogue; hence, any numerical similarity between *T*_e_ and actual planetary temperatures measured at, below or above the surface must be viewed as a coincidence. Consequently, all estimates of GE (ATE) based on Eq. (3) are misleading, since they are products of comparisons between Earth’s *observed* average surface air temperature (*T*_s_) and some *unmeasurable* (non-physical) effective radiating temperatures (*T*_e_) at the TOA. A proper assessment of ATE requires a reliable estimate of the planet’s mean global *surface* temperature in the *absence* of atmosphere. Thus, there is a practical need for a new analytic model that accounts for Hölder’s inequality while accurately predicting the average physical temperatures of airless spherical bodies.

## Methods

### Derivation of an analytic model for the mean physical temperature of airless bodies

In order to derive a formula for *T*_na_ that conforms to Hölder’s inequality, we adopted the following reasoning. The equilibrium temperature *T*_*i*_ at a point *i* on the surface of an airless planet is determined by the incident solar flux and the local surface albedo during daytime, and by the upward heat flux emanating from the regolith at night. The nighttime release of heat from the ground is assumed to primarily originate from stored solar energy in the regolith with negligible contribution by geothermal sources (e.g. Vasavada et al. 2012). Hence, the nighttime heat flux can be approximated as a *fraction* of the solar radiation absorbed by the surface during daylight hours. A robust physical model of the average surface temperature of airless bodies must also include the small effect of geothermal fluxes and cosmic microwave background radiation (CMBR). The latter only becomes important under a low solar irradiance, i.e. for ASCOs orbiting at the outskirts of the solar system. Assuming a spatial uniformity of the sub-solar (normal) albedo across the planet surface and allowing the point albedo to vary with solar incidence angle, we can write the following general equation for *T*_*i*_ using the SB law:

5 $$T_{i}=\left\{\begin{array}{ccc} {\left[\frac{\left(1-\eta \right)\;S_{o\;}\left[1-A\left(\theta_{i}\right)\right]\cos\theta_{i}+\left(R_{C}+R_{g}\right)}{\varepsilon \;\sigma }\right]}^{1/4}\; & \mathrm{if} & 0\;\leq \;\theta_{i}\;<\frac{\pi }{2}\\ {\left[\frac{\left(R_{C}+R_{g}\right)-\eta \;S_{o\;}\left[1-A\left(\theta_{i}\right)\right]\cos\theta_{i}}{\varepsilon \;\sigma }\right]}^{1/4} & \mathrm{if} & \frac{\pi }{2}\;\leq \;\theta_{i}\;\leq \;\pi \end{array}\right.$$

Here, *S*_*o*_ is the solar (stellar) irradiance (W m^−2^), *θ*_*i*_ is the incidence angle of shortwave radiation (rad) at point *i* (i.e. the angle between stellar rays and an axis normal to the surface at that point), *A*(*θ*_*i*_) is the albedo as a function of *θ*_*i*_, *η* is the fraction of absorbed solar flux stored into regolith through heat conduction, *R*_*c*_ = *σ* 2.725^4^ = 3.13 × 10^−6^ W m^−2^ is CMBR (Fixsen 2009), *R*_*g*_ is a spatially uniform geothermal flux (W m^−2^), and *ε* is the average regolith long-wave emissivity; typically 0.95 ≤ *ε* < 0.99; in this study *ε* = 0.98. The upper portion of Eq. (5) describes the surface temperature at point *i* during daytime, while the lower portion defines the respective temperature at night. During daylight hours when the Sun is above the horizon (0 ≤ *θ*_*i*_ ≤ *π*/2), the solar radiation absorbed at point *i* is partitioned into a flux giving the surface its daytime temperature, i.e. (1 − *η*) *S*_*o*_ [1 − *A*(*θ*_*i*_)]cos *θ*_*i*_, and another smaller flux conducted and stored into the ground as heat. At night, when the Sun is below the horizon (*π*/2 ≤ *θ*_*i*_ ≤ *π*), the stored heat is released giving the surface point its nighttime temperature; hence, the presence of the term *η S*_*o*_ [1 − *A*(*θ*_*i*_)]cos *θ*_*i*_ in the nighttime portion of Eq. (5). Since CMBR is virtually isotropic, we assume *R*_*c*_ to be uniformly absorbed by the daytime and nighttime hemisphere of an airless body. Note that Eq. (5) only describes location-specific differences in point equilibrium temperatures and does not simulate temporal temperature changes. This is so, because our objective is to conduct spatial integration and derive a *spherical* temperature average.

**Figure 2:**
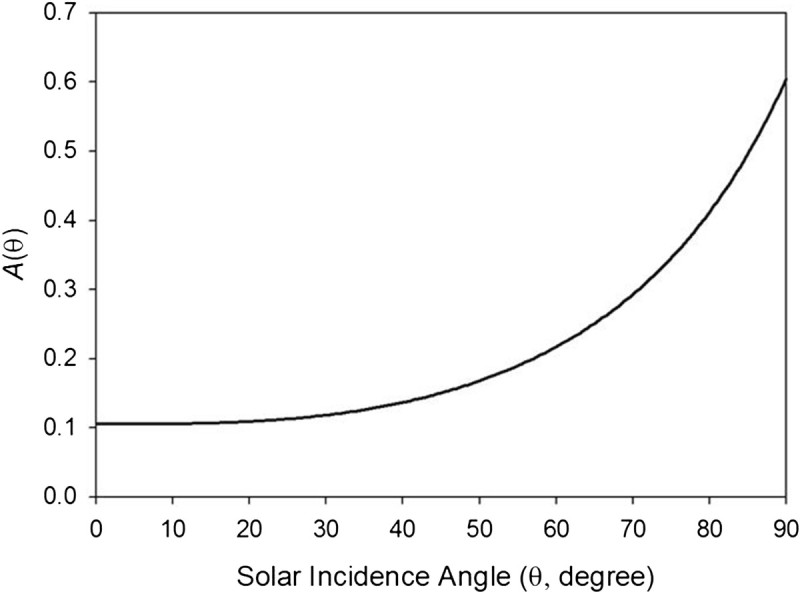
**Variation of the moon’s regolith albedo** ***A*** **(** ***θ*** **)** **as a function of solar incidence angle according to Eq. (** 6**) based on surface measurements by Apollo and Diviner missions (Keihm** 1984**; Vasavada et al.**^2012^**).**

The Moon is our serendipitous ASCO example. In situ lunar measurements by the Apollo Mission and remote observations by the NASA Diviner Lunar Radiometer Experiment (DLRE) suggest that the albedo of regolith-covered surfaces in a vacuum varies with solar incidence angle according to the function (Keihm 1984; Vasavada et al. 2012):

6 $$A\left(\theta_{i}\right)=A_{o}+0.045{\left(\theta_{i}/45\;\right)}^{3}+0.14{\left(\theta_{i}/90\;\right)}^{8}$$

where *θ*_*i*_ is in degree and *A*_*o*_ is the normal (sub-solar) albedo at *θ*_*i*_ = 0°. Figure 2 depicts the response of *A*(*θ*_*i*_) to variations in the solar angle assuming *A*_*o*_ = 0.105, the average normal albedo suggested by Diviner equatorial measurements (Vasavada et al. 2012).

Upon substituting *μ* ≡ cos *θ*_*i*_ in Eq. (5), the average global surface temperature of an airless celestial body *T*_na_ (K) is obtained from the spherical integration of *T*_*i*_, i.e.

7 $$T_{na}\;=\;\frac{1}{4\pi }\;\int_{0}^{2\pi }\left(\int_{-1}^{1}T_{i}\;d\mu \right)\;d\varphi$$

**Figure 3:**
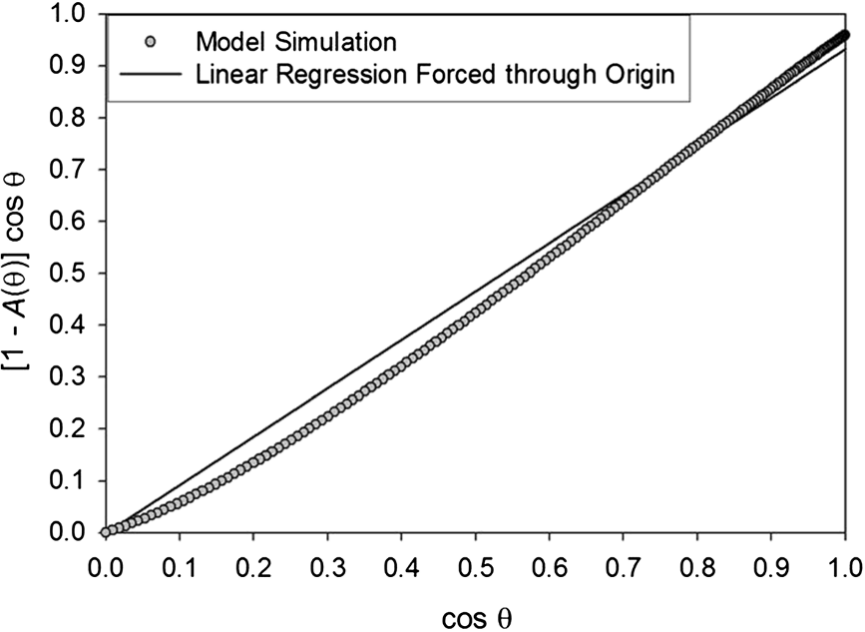
**Emergent linear relationship between cos** ***θ*** **and the regolith shortwave absorption term [1 −** ***A*** **(** ***θ*** **)] cos** ***θ*** **, where** ***θ*** **is the solar incidence angle and** ***A*** **(** ***θ*** **) is the surface albedo as a function of** ***θ*** **defined by Eq. (** 6**) (see Figure**2**).**

Inserting the albedo function (6) into Eq. (5), however, renders integral (7) without a closed-form solution. To resolve this we plotted the absorption term [1 − *A*(*θ*_*i*_)] cos *θ*_*i*_ in Eq. (5) versus the integration variable cos *θ*_*i*_. The result is a monotonic relationship that can closely be approximated by a linear regression forced through the origin (Figure 3), i.e.

8 $$\left[1-A\left(\theta_{i}\right)\right]\cos\theta_{i}\approx s_{\theta }\cos\theta_{i}$$

with *s*_*θ*_ being the regression slope. Equation (8) implies (1 − *α*_*e*_) = *s*_*θ*_, where *α*_*e*_ is an *effective* surface albedo that incorporates the impact of a variable *A*(*θ*_*i*_) on the surface temperature in the context of Eq. (5) and its spherical integral (7). Further analysis employing a range of values for *A*_*o*_ in Eq. (6) reveals:

9 $$\alpha_{e}\approx A_{o}+0.026$$

Equation (9) yields *α*_*e*_ = 0.131 for the Moon according to Diviner observations. As discussed below, the heat storage fraction *η* also varies with latitude. Thus, as with the albedo, it is more appropriate to use an *effective* heat storage fraction (*η*_*e*_) in Eq. (5) rather than *η*.

The above transformations allow us to employ a fixed albedo (*α*_*e*_) in Eq. (5) and solve integral (7) analytically to obtain a closed-form expression for *T*_na_, i.e.

10 $$\begin{array}{ll} T_{na} & =\frac{1}{4\pi }\int_{0}^{2\pi }\left\{\overset{1}{\underset{0}{\int }}{\left[\frac{\;\left(1-\eta_{e}\right)\;S_{o\;}\left(1-\alpha_{e}\right)\;\mu +R_{c}+R_{g}}{\varepsilon \sigma }\right]}^{1/4}\;d\mu \right.\\ \;\left.+\int_{-1}^{0}{\left[\frac{R_{c}+R_{g}-\eta_{e}\;S_{o\;}\left(1-\alpha_{e}\right)\mu }{\varepsilon \sigma }\right]}^{1/4}d\mu \right\}d\varphi \\ =\frac{1}{4\pi \;}\int_{0}^{2\pi }\left\{\frac{4}{5}\frac{{\left[\left(1-\eta_{e}\right)\;S_{o\;}\left(1-\alpha_{e}\right)+R_{c}+R_{g}\right]}^{5/4}-{\left(R_{C}+R_{g}\right)}^{5/4}}{\left(1-\eta_{e}\right)S_{o\;}\left(1-\alpha_{e}\right){\left(\varepsilon \sigma \right)}^{1/4}}\right.\;\\ \;\left.+\frac{4}{5}\frac{{\left[\eta_{e}\;S_{o\;}\left(1-\alpha_{e}\right)+R_{c}+R_{g}\right]}^{5/4}-{\left(R_{C}+R_{g}\right)}^{5/4}}{\eta_{e}S_{o\;}\left(1-\alpha_{e}\right){\left(\varepsilon \sigma \right)}^{1/4}}\right\}d\varphi \\ =\frac{2}{5}\left\{\frac{{\left[\left(1-\eta_{e}\right)\;S_{o\;}\left(1-\alpha_{e}\right)+R_{c}+R_{g}\right]}^{5/4}-{\left(R_{C}+R_{g}\right)}^{5/4}}{\left(1-\eta_{e}\right)\;S_{o\;}\left(1-\alpha_{e}\right){\left(\varepsilon \sigma \right)}^{1/4}}\right.\\ \;\left.+\frac{{\left[\eta_{e}\;S_{o\;}\left(1-\alpha_{e}\right)+R_{c}+R_{g}\right]}^{5/4}-{\left(R_{C}+R_{g}\right)}^{5/4}}{\eta_{e}\;S_{o\;}\left(1-\alpha_{e}\right){\left(\varepsilon \sigma \right)}^{1/4}}\right\} \end{array}$$

A numerical analysis of the final equation (10) reveals that the effect of CMBR on *T*_na_ is negligible for *S*_*o*_ > 0.15 W m^−2^. In addition, the impact of geothermal fluxes on the surface temperature of airless bodies is oftentimes insignificant. Thus, in most cases, the above formula can be simplified by substituting *R*_*c*_ = *R*_*g*_ = 0. This produces:

11a $$T_{na}=\frac{2}{5}{\left[\frac{S_{o\;}\left(1-\alpha_{e}\right)}{\varepsilon \sigma }\right]}^{0.25}\Phi \left(\eta_{e}\right)$$

where *Φ*(*η*_*e*_) ≥ 1.0 is given by:

11b $$\Phi \left(\eta_{e}\right)=\;{\left(1-\eta_{e}\right)}^{0.25}+{\eta_{e}}^{0.25}$$

The complete formula (10) only needs to be used if *S*_*o*_ ≤ 0.15 W m^−2^ and/or *R*_*g*_ is significant compared to *S*_*o*_. This is because, as *S*_*o*_ → 0.0, Eq. (11a) approaches 0.0 as well, while Eq. (10) approaches 2.73 K, the irreducible minimum temperature of deep Space assuming *ε* = 0.98.

**Figure 4:**
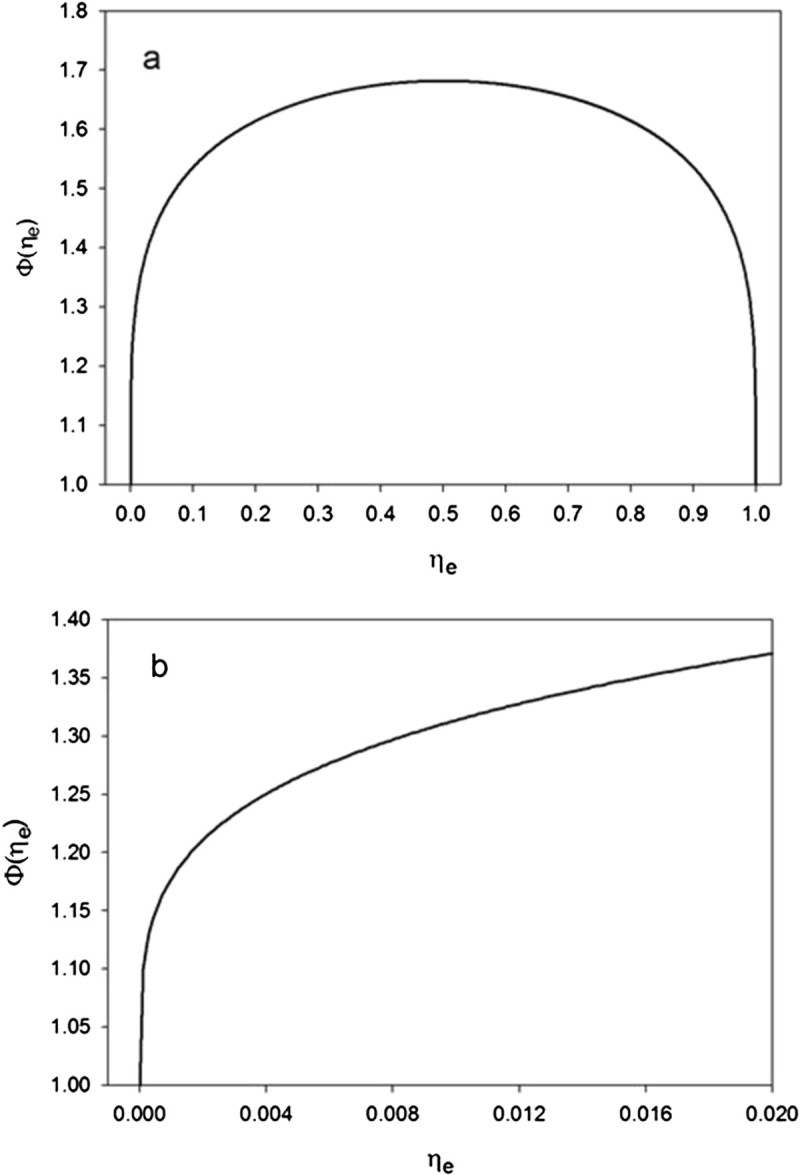
**The planet thermal enhancement factor (Φ** **) as a function of the effective regolith heat storage fraction (** ***η***_***e***_ **) according to Eq. (** **11b** **): a)** **over the entire theoretical range of** ***η***_***e***_ **;** **b)** **over the physically feasible range of** ***η***_***e***_ **.**

Conceptually *Φ*(*η*_*e*_) is a non-dimensional thermal enhancement factor that boosts the global temperature of an airless planet above the level expected from a surface with zero thermal inertia, i.e. if the planet were completely non-conductive to heat. Thanks to *η*_*e*_ > 0, the night sides of rotating ASCOs remain at a significantly higher temperature than expected from CMBR alone. This substantially raises the global average temperature of ASCOs compared to the case when *η*_*e*_ = 0. In theory, *η*_*e*_ can vary in the interval 0.0 ≤ *η*_*e*_ ≤ 1.0. However, due to physical constraints imposed by the low thermal conductivity of regolith in an airless environment, this range is considerably narrower in reality. For actual ASCOs, we expect 0.005 < *η*_*e*_ < 0.02 based on thermal conductivity data for the lunar regolith reported by Vasavada et al. (2012). Figure 4a illustrates the response of *Φ*(*η*_*e*_) to variation in *η*_*e*_ over the entire theoretical range, while Figure 4b depicts the same response over the approximate physically feasible range 0.0 ≤ *η*_*e*_ ≤ 0.02. According to Eq. (11b), *Φ*(*η*_*e*_) reaches a maximum of 1.682 at *η*_*e*_ = 0.5. However, since it is not possible for a regolith immersed in vacuum to store on average as much as 50% of the absorbed solar energy as heat, *Φ*(*η*_*e*_) cannot practically ever reach its theoretical maximum. Realistically, we expect 1.26 < *Φ*(*η*_*e*_) < 1.37 for ASCOs.

Equation (11a) is similar to Eq. (15) in Rubincam (2004) and to Equation four in Leconte et al. (2013) except for the additional temperature enhancement factor *Φ*(*η*_*e*_) that was not considered by these researchers. Previous studies have also not addressed the physical incompatibility between ASCO’s actual average surface temperature and its effective emission temperature computed from Eq. (3). This incompatibility is revealed in our derivation by the following comparison. Using *S*_*o*_ = 1,360.9 W m^−2^, *α*_*e*_ = 0.131 and *ε* = 0.98 in Eq. (3) yields an effective emission temperature for the Moon *T*_e_ = 270.1 K. This estimate is 13.2 K *higher* than the theoretically maximum possible temperature *T*_na_ = 256.9 K produced by Eq. (11a) using the same input and a physically unreachable peak value of *Φ*(*η*) = 1.682 corresponding to *η*_*e*_ = 0.5. Thus, in the absence of a significant geothermal flux, it is *in principle* not possible for an airless body to reach an average global temperature as high as its effective emission temperature.

### Verification of the new analytic temperature model for airless bodies

Since Eq. (10) and its simplified form (11a) were derived to predict the average surface temperature of celestial bodies with no tangible atmospheres, it is prudent to verify them against data for the Moon as the closest and best-studied airless object in the Solar System. Lunar temperatures have been measured for more than 50 years both remotely via Earth-based telescopes and instruments aboard lunar orbiters, and in situ by the Surveyor and Apollo landing mission (Paige et al. 2010a). Recently, the Diviner Lunar Radiometer Experiment (Paige et al. 2010a), a part of the Lunar Reconnaissance Orbiter (LRO) Mission (Vondrak et al. 2010), launched an extensive remote-sensing survey of the lunar surface. The Diviner instrument aboard LRO provides measurements in two spectral channels of reflected shortwave radiation and seven channels of emitted infrared long-wave radiation (Paige et al. 2010a; Vasavada et al. 2012). The goal of DLRE is to map the Moon’s surface temperature and albedo at a high spatial and temporal resolution over multiple diurnal and seasonal cycles. DLRE is the most comprehensive attempt to date to quantify the spatial and temporal variability of the lunar surface temperature. Although the project is still in progress, data acquired since the beginning of DLRE’s commissioning period (the summer of 2009) already cover most of the Moon surface. These high-quality radiance measurements have been utilized to study thermal environments at the lunar equator, mid-latitudes and the Polar Regions, and to validate and refine existing thermo-physical models (Paige et al. 2010b; Bandfield et al. 2011; Vasavada et al. 2012).

#### Verification approach

In order to obtain an independent estimate of the Moon’s mean surface temperature needed to verify Equations (10) and (11a) we employed a detailed NASA thermo-physical model of the regolith called TWO that has been previously verified against Apollo multi-year borehole measurements on the Moon and remote-sensing observations of Mercury (Vasavada et al. 1999). The name TWO originates from the two layers used in early versions of the model to describe an assumed abrupt change in the regolith thermo-physical properties with depth. Recently Vasavada et al. (2012) revised the model by allowing both thermal conductivity and bulk density to gradually increase with depth. The updated model accurately reproduced 513,738 Diviner temperature measurements along the lunar Equator taken over a period of 2.5 years and covering 4 complete diurnal lunar cycles (Vasavada et al. 2012). TWO uses ‘first principles’ to simulate point-level surface energy balance and a 1-D subsurface heat flow. The model calculates subterranean transport and storage of heat as a function of depth-varying thermal conductivity and bulk density of the lunar regolith.

We chose a validated physics-based model to verify Equations (10) and (11a) over the actual Diviner measurements, because the latter do not yet provide the temporal and global spatial coverage necessary for a robust estimation of the Moon mean annual equilibrium surface temperature. In addition, the Diviner data set literally contains millions of raw radiometric measurements that require a dedicated project to properly screen and convert into temperature readings. Since TWO has been shown to accurately reproduce Diviner-measured temperatures under a wide range of conditions and the Moon’s regolith appears to be spatially highly homogeneous in terms its thermo-physical properties (Vasavada et al. 2012), we assumed that this model would yield sufficiently accurate results for all lunar locations.

**Figure 5:**
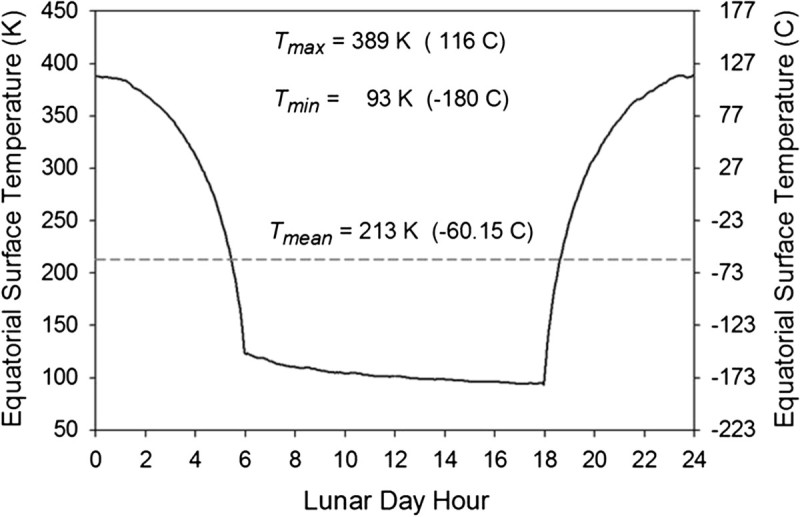
**Typical diurnal course of the Moon’s equatorial surface temperature according to Diviner radiometric measurements and simulations by the revised TWO model (based on data in Figure nine(a) of Vasavada et al.** 2012**).** Also shown are the maximum (*T*_*max*_), minimum (*T*_*min*_) and mean (*T*_*mean*_) temperature of the lunar Equator. In agreement with Hölder’s inequality, *T*_*mean*_ = 213 K is about 57 K cooler than the Moon’s effective emission temperature (270.2 K) calculated from Eq. (3).

Figure 5 depicts the average diurnal course of surface temperature at the lunar Equator simulated by the revised TWO model. As illustrated in Figure nine of Vasavada et al. (2012), this temperature curve agrees quite well with hundreds of thousands of Diviner measurements. The curve yields a mean equatorial temperature of 213 K (−60.15 C). In accordance with Hölder’s inequality, the warmest latitude on the Moon is on average 57.1 K cooler than the lunar effective emission temperature (≈270 K) computed from Eq. (3).

We employed a three-step approach to obtain an independent estimate of the Moon’s mean annual global surface temperature. First, we ran the TWO model as described by Vasavada et al. (2012) at every 5 degree latitude from the lunar Equator to the Poles calculating an annual-mean temperature for each latitude that represented both Northern and Southern lunar Hemisphere. The simulation employed a temporal resolution of 0.01 lunar hours and actual orbital characteristics of the Moon derived from publically available ephemerides of the Navigation and Ancillary Information Facility at the Jet Propulsion Laboratory. Solar irradiance was set to *S*_*o*_ = 1,360.9 W m^−2^ at a distance of 1 AU based on recent satellite observations reported by Kopp and Lean (2011). The irradiance was allowed to vary during the course of a year in accordance with Earth’s small orbital eccentricity. The shortwave albedo of regolith was modeled as a function of the solar incidence angle using Eq. (6) with the normal albedo set to *A*_*o*_ = 0.105 based on Diviner observations (Vasavada et al. 2012). The thermal emissivity of regolith was assumed to be spatially invariant and equal to 0.98 (Vasavada et al. 2012). TWO was run for all 5-degree latitude bands over multiple years to allow equilibration of the annual-mean temperatures.

Next we fitted a 6th order polynomial to the modeled latitudinal temperature averages to derive a continuous function that smoothly describes the variation of the lunar annual-mean temperature *T* (K) with latitude *L* (rad):

12 $$\begin{array}{ll} T\left(L\right)= & 212.9+9.919L-119.814L^{2}\\ +307.116L^{3}-466.244L^{4}\\ +321.317L^{5}-84.973L^{6} \end{array}$$

**Figure 6:**
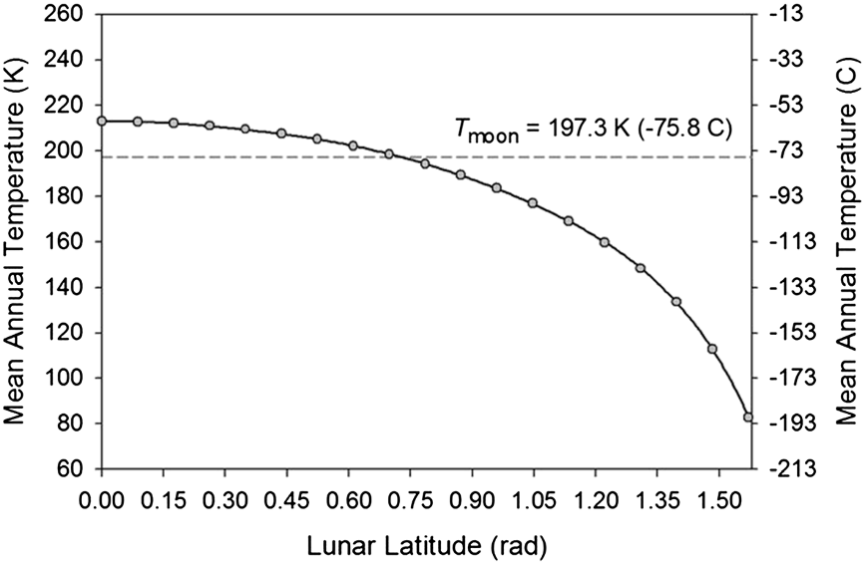
**Mean annual temperature of the lunar surface as a function of latitude according to results from the validated TWO model (Vasavada et al.** 1999**,**^2012^**) (black dots).** The smooth curve represents a 6th-order polynomial (Eq. 12) fitted through the latitudinal temperature averages via a least-squares regression. The Moon mean annual global temperature, *T*_*moon*_ = 197.3 K (marked by a horizontal dashed line) was estimated through integration of the polynomial (12) using formula (13).

Finally, the Moon’s global mean temperature (*T*_*moon*_) was calculated via integration of *T*(*L*), i.e.

13 $$T_{\mathrm{moon}}=\int_{0}^{\pi /2}T\left(L\right)\cos L\;\mathrm{dL}$$

where cos *L* is a polar coordinate area-weighting factor. Equation (13) yielded *T*_*moon*_ = 197.3 K. To our knowledge, this is the first physically robust estimate of the Moon’s true average global surface temperature reported in the scientific literature. Figure 6 displays the results from the above computational approach. Note that the entire lunar latitudinal temperature curve lies well below the 270.1 K effective emission temperature derived from Eq. (3) with *T*_*moon*_ being nearly 73 K cooler than *T*_e_. This illustrates the physical incompatibility between *T*_e_ and *T*_*moon*_, which is mathematically explained by Hölder’s inequality.

#### Verification results

**Figure 7:**
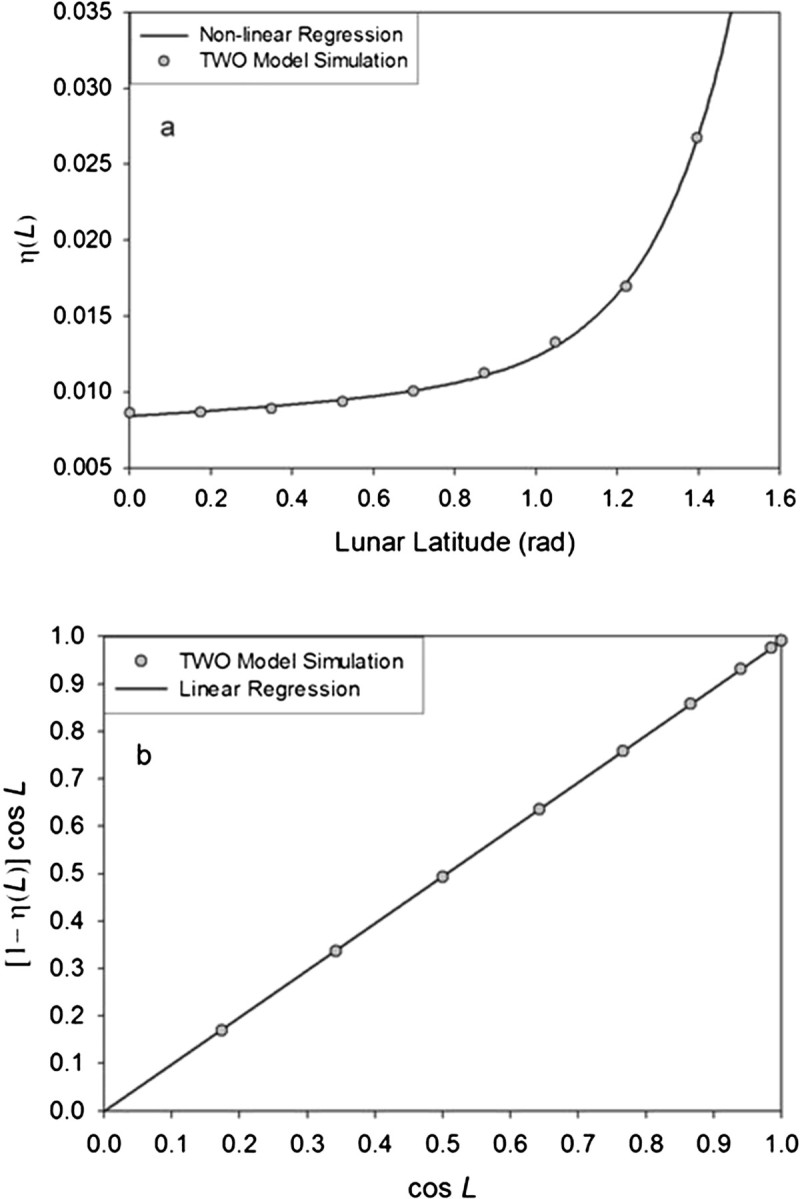
**Relationship between the heat storage fraction of lunar regolith (** ***η*** **) and latitude (** ***L*** **): a)** variation of  as a function of *L* according to the TWO thermo-physical model output (Vasavada et al. 2012); **b)** emergent linear relationship between $\left[1-\eta \left(L\right)\right]\cos L$ and cosL.

In order to properly verify the new model against the above independent estimate of the Moon mean surface temperature we used equivalent values for the driving variables in the formulas (10) and (11a) to those employed in the TWO thermo-physical model, i.e. *S*_*o*_ = 1,360.9 W m^−2^ and *ε* = 0.98. The effective shortwave albedo was set to *α*_*e*_ = 0.131 according to Eq. (9). To obtain an estimate of the effective heat storage fraction (*η*_*e*_) in Eq. (10) we analyzed output from the TWO model. First, we computed the annual fraction of the absorbed solar flux conducted into regolith (*η*) at several latitudes in order to evaluate its meridional variation. Latitudinal *η* values were calculated as ratios of the cumulative outgoing nighttime heat fluxes to the total daily-absorbed solar fluxes over the course of a typical lunar year. We found that *η*(*L*) increases non-linearly with latitude (Figure 7a). Such a functional relationship cannot be directly incorporated into equations (10) and (11a), since the integral formula calls for a *single* effective value *η*_*e*_. To estimate the latter we plotted [1 − *η*(*L*)] cos *L* versus cos *L* to discover a tight linear relationship between these variables with virtually zero intercept (Figure 7b). Since in the context of spherical integration, both cos *L* and cos *θ* vary over the same numerical range (0 – 1), the term [1 − *η*(*L*)] cos *L* is equivalent to (1 − *η*) cos *θ* in the daytime portion of Eq. (5). Hence, one can use the slope *s* of the linear regression in Figure 7b to calculate an *effective* heat storage fraction *η*_*e*_ for Eq. (10). Specifically, the equality [1 − *η*(*L*)] cos *L* = *s* cos *L* implies (1 − *η*_*e*_) = *s*, which effectively neutralizes the meridional variation of the heat storage fraction *η*(*L*) in the daytime portion of Eq. (5). Since *s* = 0.99029 (Figure 7b), we obtain *η*_*e*_ = 1 − *s* = 0.00971. Hence, the lunar surface effectively stores about 1% of the absorbed solar flux into the regolith as heat. This energy is subsequently released at night giving the dark side of the Moon a significantly higher surface temperature than expected from the cosmic background radiation alone.

Using the above values of *S*_*o*_, *α*_*e*_, *η*_*e*_ and *ε* in either Eq. (10) or (11a) produces *T*_na_ = 200.4 K for the mean surface temperature of the Moon. This estimate is 3.1 K higher than *T*_*moon*_ = 197.3 K inferred from the TWO model (Eq. 13). However, considering the 72.8 K difference between *T*_e_ calculated from Eq. (3) and *T*_*moon*_, it appears that equations (10) and (11a) provide a much more accurate estimate of the Moon average surface temperature compared to Eq. (3). Based on Hölder’s inequality, we expect this to be the case for any ASCO. It is worth noting that Eq. (15) in Rubincam (2004) and Equation four in Leconte et al. (2013), which are similar to our Eq. (11a) without the heat storage term *Φ*(*η*_*e*_), yield a 44.5 K lower global Moon temperature than *T*_*moon*_. This demonstrates the critical importance of *Φ*(*η*_*e*_). Without this enhancement factor our analytic model would have failed the verification against NASA Moon temperature data. Nevertheless, it is informative to explore the reasons for the small discrepancy between *T*_na_ predicted by Eq. (11a) and *T*_*moon*_.

**Figure 8:**
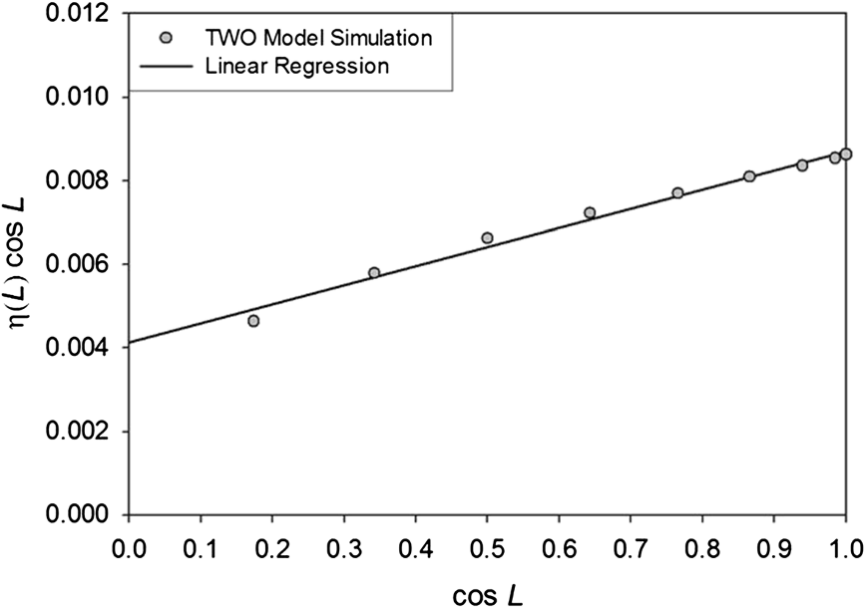
**Emergent linear relationship between** ***η*** **(** ***L*** **)** **cos** ***L*** **and** **cos** ***L*** **based on modeled data in Figure**7***a*** **.**

A close examination of Eq. (5) reveals that its nighttime portion contains the product *η* cos *θ*, which is numerically equivalent to *η* cos *L* in the context of spatial integration. If *η* cos *L* is plotted versus cos *L* using data from Figure 7a, a linear relationship emerges (Figure 8) similar to one in Figure 7b, but with an intercept that is significantly different from zero, i.e. *η* cos *L* = *a* cos *L* + *b*, where *a* = 0.00458 and *b* = 0.00413. This means that the term *η* cos *θ* in the nighttime portion of Eq. (5) effectively linearizes the variation of *η* with latitude (Figure 7a) in the context of integral (7), but does not completely neutralize it as achieved by the term (1 − *η*) cos *θ* in the daytime portion of the same equation (Figure 7b). Hence, *η*_*e*_ derived from daytime conditions does not have the exact same mathematical meaning and magnitude in the nighttime portion of Eq. (10). Indeed, it can be shown that integrating the nighttime portion of Eq. (5) upon substituting *η* cos *L* with *a* cos *L* + *b* yields a complex solution. In other words, the slight overestimation of the Moon global surface temperature by equations (10) and (11a) appears to be the result of using an *η*_*e*_ value in the nighttime portion of the formula derived from a relationship that is strictly valid for daytime conditions (Figure 7b).

### Refinement of the analytic temperature formula for airless bodies

The above analysis suggests the need to adjust *η*_*e*_ in the nighttime portion of Eq. (10) in order for the analytic model to more accurately predict average global temperatures. Using data from Figure 8, we estimated a compensation factor 0.754 for *η*_*e*_ in the nighttime portion of Eq. (10). T model:

14 $$\begin{array}{ll} T_{na}=\;\frac{2}{5} & \left\{\frac{{\left[\left(1-\eta_{e}\right)\;S_{o\;}\left(1-\alpha_{e}\right)+R_{c}+R_{g}\right]}^{5/4}-{\left(R_{C}+R_{g}\right)}^{5/4}}{\left(1-\eta_{e}\right)\;S_{o\;}\left(1-\alpha_{e}\right){\left(\varepsilon \sigma \right)}^{1/4}}\right.\\ \;\left.+\frac{{\left[0.754\;\eta_{e}\;S_{o\;}\left(1-\alpha_{e}\right)+R_{c}+R_{g}\right]}^{5/4}-{\left(R_{C}+R_{g}\right)}^{5/4}}{0.754\;\eta_{e}\;S_{o\;}\left(1-\alpha_{e}\right){\left(\varepsilon \sigma \right)}^{1/4}}\right\} \end{array}$$

Similar to Eq. (10), here one can also safely assume *R*_*c*_ = 0.0 if *S*_*o*_ > 0.15 W m^−2^ and *R*_*g*_ = 0.0 in most cases. This reduces Eq. (14) to (11a) with the regolith thermal enhancement factor redefined as:

15 $$\Phi \left(\eta_{e}\right)=\;{\left(1-\eta_{e}\right)}^{0.25}+0.932\;{\eta_{e}}^{0.25}$$

where 0.932 = 0.754^0.25^. Thus, we now arrive at a simple yet robust and sufficiently accurate analytic expression for calculating the average surface temperatures of airless celestial bodies when *S*_*o*_ > 0.15 W m^−2^:

16 $$T_{na}=\frac{2}{5}{\left[\frac{S_{o\;}\left(1-\alpha_{e}\right)}{\varepsilon \sigma }\right]}^{0.25}\left[{\left(1-\eta_{e}\right)}^{0.25}+0.932\;{\eta_{e}}^{0.25}\right]$$

In rare cases, where *S*_*o*_ ≤ 0.15 W m^−2^, one must use formula (14) instead. Equation (14) is also recommended for airless bodies with a significant contribution of heat to the surface (*R*_*g*_) by geothermal sources. Equation (16) yields 197.1 K for the Moon, a value within the uncertainty of the best estimate derived from the TWO thermo-physical model.

We hypothesize that regolith-covered ASCOs would have similar effective albedos and heat storage coefficients because, in the absence of atmosphere, the pulverization of surface materials by micrometeoroids and cosmic radiation becomes the predominant geologic process creating a top substrate of similar particle-size distribution and thermo-physical properties. Hence, it might be reasonable to employ the Moon-based value of *η*_*e*_ = 0.00971 in estimating the average surface temperatures of other airless bodies such as Mercury, for example. Using a solar irradiance *S*_*o*_ = 9,086.7 W m^−2^ (corresponding to Mercury’s average distance of 0.387 AU to the Sun) and a plausible albedo range 0.068–0.142 (Mallama et al. 2002) in Eq. (16) yields 315.8 ≤ *T*_na_ ≤ 322.4 K for that planet. Note that this estimate is ~120 K lower than the one derived from Eq. (3) (440 K) and currently quoted as Mercury’s ‘average temperature’ (Williams 2014). Planetary science should soon be able to verify our prediction of Mercury’s mean surface temperature using remote infrared measurements provided by the NASA MESSENGER robotic spacecraft.

## Results and discussion

### Aggregation errors of the effective emission temperature formula

The above discussion reveals that effective emission temperatures calculated from Eq. (3) tend to be significantly higher than any long-term temperature averages on the surface of ASCOs. This discrepancy is mathematically explained by Hölder’s inequality (4b). Having derived a new theoretically robust formula for the mean surface temperatures of ASCOs, we can now quantify the aggregation errors inherent in the standard formula (3).

We note that *T*_e_ and *T*_na_ have similar functional forms, i.e. both temperatures depend on the 4th root of solar irradiance. Hence, each formula can be written as $T=c\;S_{0}^{0.25}$, where *c* is a bulk coefficient combining surface albedo, infrared emissivity, regolith heat storage, and the SB constant. Denoting such bulk coefficients as *c*_*e*_ and *c*_na_ in equations (3) and (16) respectively, we obtain:

17 $$c_{e}={\left[\frac{1-\alpha_{e}}{4\;\varepsilon \;\sigma }\right]}^{0.25}$$

and

18 $$c_{na}=\frac{2}{5}{\left[\frac{1-\alpha_{e}}{\varepsilon \;\sigma }\right]}^{0.25}\left[{\left(1-\eta_{e}\right)}^{0.25}+0.932\;{\eta_{e}}^{0.25}\right]$$

This consolidation of variables allows us to define two error functions, *E*_r_ (%) and *E*_a_ (K) quantifying the relative and absolute errors of Eq. (3) respectively, i.e.

19 $$E_{r}\;=\frac{T_{e}-T_{na}}{T_{na}}100=\frac{c_{e}-c_{na}}{c_{na}}100$$

20 $$E_{a}=T_{e}-T_{na}=\left(c_{e}-c_{na}\right)\;S_{0}^{0.25}$$

In order to evaluate *E*_r_ and *E*_a_ we employ radiative and thermo-physical data for the Moon. Note that the particular choice of parameter values is not important as long as both formulas (17) and (18) use the same ones. Upon substituting *α*_*e*_ = 0.13, ε = 0.98, and *η*_*e*_ = 0.00971 in equations (17) through (20), we obtain *c*_*e*_ = 44.48, *c*_na_ = 32.45, *E*_r_ = 37.1 % and $E_{a}=12.03\;S_{0}^{0.25}$. A greater heat storage fraction resulting from a higher thermal conductivity of the regolith would produce a larger *c*_na_, thus boosting *T*_na_ towards *T*_e_. However, based on the most likely upper limit of *η*_*e*_ ≈ 0.02 for ASCOs, we estimate the minimum errors of the emission-temperature formula to be in the order of *E*_r_ = 31.4 % and $E_{a}=10.6\;S_{0}^{0.25}$.

The above analysis reveals that Eq. (3) overestimates the average surface temperatures of ASCOs by about 37% with the absolute error of *T*_e_ increasing proportionally to the 4th root of solar irradiance (Eq. 20). Hence, *T*_e_ and *T*_na_ are not physically comparable. The numerical bias of *T*_e_ becomes particularly evident when comparing ASCOs orbiting at different distances from the Sun. For example, according to Eq. (3), Mercury (at 0.387 AU) should be about 172 K warmer than the Moon (at 1 AU). However, the theoretically correct formula (16) indicates an average temperature difference of no more than 125 K between the two bodies. Hence, Eq. (3) is not suitable for comparing the thermal regimes of planets as suggested by Leconte et al. (2013). Viewed across a range of planetary environments, the effective emission temperature shows no meaningful relationship to actual surface temperatures of bodies either with or without an atmosphere. In view of these results, we propose equations (14) and (16) as a new analytic standard for predicting the mean surface temperatures of airless spherical bodies.

### Moon as a natural airless equivalent of earth

Our approach to evaluating Earth’s total ATE rests on the supposition that, without an atmosphere, our Planet would be on average as cold as the Moon. Verifying this assumption requires addressing the following questions: 1) would the Earth surface in the absence of atmosphere have the same radiative and thermo-physical properties as the lunar regolith? and 2) is an airless Earth thermally equivalent to a hypothetical Earth with an atmosphere devoid of greenhouse gases? In other words, is ATE fully explainable by the radiative effect of heat-absorbing gases? Equations (14) and (16) provide a suitable framework for investigation. There are 4 variables in Eq. (16) impacting the average surface temperature of ASCOs: solar irradiance (*S*_*o*_), shortwave albedo (*α*_*e*_), surface long-wave emissivity (*ε*), and the regolith’s effective heat storage coefficient (*η*_*e*_).

Since Moon and Earth orbit the Sun at the same distance, they receive equal amounts of solar radiation and have the same *S*_*o*_. Serendipitously, the Moon effective albedo *α*_*e*_ = 0.131 nearly equals Earth’s present surface average cloudless albedo (0.122–0.13) inferred from satellite- and ground-based observations (Stephens et al. 2012; Wild et al. 2013). This is in spite of the fact that our Planet has highly reflective regions such as deserts, glaciers, and Polar Ice Caps that are absent on the Moon. However, the high reflectivity of these Earth surfaces is counterbalanced by the low albedo of the World’s Oceans. Aside from this coincidental similarity of surface albedos between present-day Earth and the Moon, one can also argue that, in the absence of atmosphere, Earth would have no liquid oceans and/or exposed glaciers, since these require an atmospheric pressure (*P*) and temperature (*T*) above the triple point of water to exist, i.e. *T* > 273.2 K and *P* > 611.73 Pa (Cengel and Turner 2004). Without an atmosphere, the surface of our planet would be subjected to the same geologic processes that presently govern regolith formation on the Moon (e.g. bombardment by cosmic radiation and micrometeorites). Hence, an airless Earth would likely have a surface soil layer of similar radiative and optical properties (shortwave albedo and long-wave emissivity) as the lunar regolith. The uncertainty of the ATE estimate associated with Earth’s airless albedo is further discussed below.

The effective heat storage fraction *η*_*e*_ is the only term among the independent variables in equations (14) and (16) that significantly differs between the Moon and present-day Earth. While lunar regolith stores on average less than 1% of the absorbed solar flux in the ground as heat, landmasses on Earth typically conduct 5%–6% of the daytime absorbed short- and long-wave radiation into the subsurface known as *soil heat flux*. Oceans store an even greater fraction due to the high thermal conductivity and volumetric heat capacity of water. On average, Earth conservatively stores between 7% and 8% of the total daytime absorbed radiation into the subsurface to be released as heat at night. Assuming *η*_*e*_ = 0.075 as an average value in Eq. (15) produces a significantly larger soil thermal enhancement factor *Φ*(0.075) = 1.47 for Earth compared to *Φ*(0.00971) = 1.29 for the Moon. This raises two additional questions: *a*) what enables the Earth surface to store substantially more heat than the lunar regolith?; and *b*) does a higher surface heat storage (i.e. a larger *η*_*e*_) make Earth a poor comparison to the Moon for the purpose of ATE evaluations? To answer these we must analyze the factors controlling *η*_*e*_.

#### Effect of surface thermal conductivity on regolith heat storage

At any given latitude, the heat-storage fraction *η* depends on the cumulative ground heat flux *G*_*o*_ (W m^−2^) absorbed during the course of a typical day. Instantaneous ground heat fluxes, in turn, are functions of the substrate apparent thermal conductivity *k* (W m^−1^ K^−1^) and the time-varying vertical temperature gradient (∂ *T*(*t*)/ ∂ *z*, K m^−1^) at the surface (e.g. Campbell 1985). Thus, *G*_*o*_ can be described at any latitude as

21 $$G_{o}=\;\left[\int_{t1}^{t2}\left(\;k\frac{\partial T\left(t\right)}{\partial z}\right)dt\right]/L_{d}$$

where *t*_1_ and *t*_2_ are the times of sunrise and sunset, respectively, and *L*_*d*_ = *t*_2_ − *t*_1_ is the day length at that latitude.

In the dynamics of heat flow, ∂*T*(*t*)/∂*z* varies throughout the day for a particular *k* as a function of the changing solar forcing at the surface. For a given radiation intensity, however, the temperature gradient tends to be inversely related to *k*, i.e. a higher thermal conductivity tends to produce smaller vertical temperature gradients, while a lower *k* results in larger ∂*T*(*t*)/∂*z*. This curtails the sensitivity of *G*_*o*_ to changes in *k* making *G*_*o*_ vary nonlinearly with conductivity albeit in the same direction. Thus, it takes a relatively large increase in *k* to produce a moderate rise in *G*_*o*_ for otherwise equal conditions. For example, results from a sensitivity test of the TWO model reported by Vasavada et al. (2012, their Figure six(a)) indicate that a 30-fold boost of the regolith thermal conductivity only causes a 5-fold increase of the emitted nighttime heat flux. Nevertheless, *k* controls the size of both *G*_*o*_ and *η*. To understand the factors controlling the magnitude of *k* it is informative to compare empirical models of thermal conductivity for the lunar regolith and Earth’s soils based on in-situ measurements. According to Vasavada et al. (2012), the thermal conductivity of the lunar surface varies with depth (*z*, m) and temperature (*T*, K) as

22 $$\begin{array}{ll} k\left(z,T\right)= & k_{d}-\left(k_{d}-k_{s}\right)\exp\left(-z/0.06\right)\\ +2.7k_{s}\;{\left(\frac{T}{350}\right)}^{3} \end{array}$$

where *k*_*s*_ = 0.0006 and *k*_*d*_ = 0.007 (W m^−1^ K^−1^) are respective conductivities at the surface and at ~0.1 m depth. The factor 2.7 is a ratio of the radiative to solid component of *k* at *T* = 350 K. The apparent thermal conductivity of a mineral soil on Earth *λ*(*w*), which includes the effects of sensible and latent heat transport, can be described by the following function (Cass et al. 1984; Campbell 1985):

23 $$\lambda \left(w\right)=A+Bw\left(1-f_{r}\right)-\left(A-D\right)\exp\left\{-{\left[Cw\left(1-f_{r}\right)\right]}^{4}\right\}$$

where *w* is the volumetric soil moisture content (m^3^ m^−3^), *f*_*r*_ is the volumetric fraction of rocks (i.e. particles with a diameter greater than 2 mm), and *A,B,C* and *D* are empirical coefficients depending on *f*_*r*_, total soil porosity (*p*_*s*_, m^3^ m^−3^), and percent clay (*C*_*l*_) in soil, so that

$$\begin{array}{l} A=\frac{1.57-p_{s}\left(1-f_{r}\right)}{1-0.52\left[1-p_{s}\left(1-f_{r}\right)\right]}-2.8p_{s}\left[1-p_{s}\left(1-f_{r}\right)\right]\\ B=2.8\left[1-p_{s}\left(1-f_{r}\right)\right]\\ C=1+26{C_{l}}^{-0.5}\\ D=0.03+0.7{\left[1-p_{s}\left(1-f_{r}\right)\right]}^{2} \end{array}$$

According to Eq. (22), at 0.5 cm depth and typical Earth temperatures (i.e. 263 K–310 K), the thermal conductivity of the lunar regolith is only 0.0018 ≤ *k* ≤ 0.0022 W m^−1^ K^−1^. For comparison, a completely dry soil of similar texture, bulk density, and rock content (i.e. *p*_*s*_ = 0.494 m^3^ m^−3^, *f*_*r*_ = 0.028, and *C*_*l*_ = 0.01) on Earth has a thermal conductivity *λ*(0) = 0.219 W m^−1^ K^−1^ according to Eq. (23). Increasing the soil moisture content to its maximum (*w* = *p*_*s*_) boosts that conductivity to *λ*(0.494) = 1.473 W m^−1^ K^−1^. Hence, a substrate of similar particle size distribution and bulk density as the lunar regolith is over 100 times more conductive to heat on Earth than it is on the Moon. A moisture-saturated soil of the same type on Earth has over 700 times greater thermal conductivity than the lunar regolith.

The immense difference in the ability to conduct heat between Moon and Earth can be explained by analyzing the components of the apparent thermal conductivity, i.e. solid, radiative, and convective (the latter component includes sensible and latent heat transport). Solid conduction results from the vibrational transfer of energy between atoms comprising the material lattice of regolith particles. This type of conduction increases with particle size and bulk density of the substrate. The radiative component arises from radiant heat exchange between regolith grains and is proportional to the third power of the grains’ absolute temperature (Vasavada et al. 2012). At relatively low bulk densities and high temperatures found near the surface, radiant heat exchange typically dominates the regolith thermal conductivity in airless environments. The convective component of heat conduction is due to collision of gas molecules residing in the space between soil particles and requires the presence of an atmosphere to operate. Since sensible and latent heat fluxes are several orders of magnitude more effective in transporting energy compared to radiation or solid conduction, the interstitial micro-convection becomes the predominant mechanism of thermal conduction in porous media immersed in an atmosphere. Indeed, laboratory experiments by Presley and Christensen (1997) have shown that the apparent thermal conductivity of dry regolith increases with the 2/3-power of atmospheric pressure between 0 Pa and 1,000 Pa. These results have recently been confirmed by in-situ measurements of Martian soil made by the Thermal and Electrical Conductivity Probe (TECP) aboard the Phoenix Lander (Zent et al. 2010). The observed surface thermal conductivity in the northern polar region of the Red Planet (~0.085 W m^−1^ K^−1^) is consistent with measurements made by Presley and Christensen (1997) in a simulated Martian atmosphere on Earth. In other words, thanks to the presence of a tangible atmosphere, Mars has a nearly 50-time higher thermal conductivity than the Moon. Hence, it is the presence of an effective vacuum and the related lack of gaseous micro-convection within the lunar regolith that makes the Moon such a poor heat conductor. This implies that regolith-covered ASCOs can be expected to have similarly low surface thermal conductivities. The current Earth surface is vastly more conductive to heat than either lunar regolith or Martian soil because of the sizable atmospheric pressure present on our planet. Earth’s thermal conductivity is further boosted by moisture (liquid water), which cannot exist without ATE. In the absence of atmosphere, there would be no interstitial convection to boost *η*_*e*_. Therefore, an airless Earth would have a surface of similar thermo-physical properties as the present lunar regolith. The strong dependence of surface thermal conductivity and *η*_*e*_ on atmospheric pressure and soil moisture lends additional physical support to the notion that Earth’s overall ATE ought to be evaluated with respect to an equivalent *airless* environment rather than a hypothetical atmosphere devoid of greenhouse gases.

#### Effect of planet’s rotation rate on regolith heat storage

Propositions have been made in the literature that a planet spin rate (*ω*, Hz) can affect the average surface temperature of ASCOs. Specifically, it has been suggested that a higher *ω* would cause a planet’s *T*_na_ to approach *T*_e_ (e.g. Smith 2008). A comprehensive mathematical analysis of the effect of rotation on surface temperature is beyond the scope of this study. Here we shall only briefly explore the expected equilibrium response of a planet’s global surface temperature to a sustained change in spin rate according to the standard theory of heat flow.

The effective heat storage fraction *η*_*e*_ is the only variable in Eq. (16) that might potentially be affected by a change in planet’s spin rate. Since *η*_*e*_ is a function of *η*(*L*) (Figure 7), first we need to investigate whether rotational speed can influence the equilibrium heat-storage coefficient *η*_*L*_ at any latitude *L*. We begin with the mathematical definition of *η*_*L*_ as a ratio of the cumulative daytime ground heat flux to the daily total absorbed solar flux adopted in this study, i.e.

24 $$\eta_{L}=\;\left\{\int_{t1}^{t2}\left(\;k\frac{\partial T\left(t\right)}{\partial z}\right)dt\right\}\;/\;\left\{\int_{t1}^{t2}S_{L}\left[1-A\left(\theta ,t\right)\right]\cos\left[\theta \left(t\right)\right]dt\right\}$$

where *S*_*L*_ is the maximum incident solar radiation at latitude *L*, *θ*(*t*) is the solar zenith angle as a function of time *t*, *A*(*θ ,t*) is the surface albedo as a function of *θ*(*t*), and (*t*_2_ − *t*_1_) is the day length. This definition assumes that, in equilibrium, all the energy conducted into regolith during daytime is completely released at night warming the surface on the dark side of the planet. Obviously, an increase of rotational speed would shorten the day length, which will reduce both the amount of absorbed daily shortwave radiation and the period of heat conduction into the regolith. However, the key question is how would a change in the spin rate affect the numerator of Eq. (24) via instantaneous heat fluxes? To answer it we analyze an idealized analytic solution to the heat flow equation discussed by Campbell (1985) and Ochsner et al. (2007).

According to the classic theory of heat flow in porous media, increasing *ω* reduces the depth to which the surface heat wave can propagate. This is mathematically described by the so-called ‘damping depth’ (*Z*_*d*_, m) as referred to in soil science (Campbell 1985) or ‘thermal skin depth’ as named in planetary science (Leyrat et al. 2011):

25 $$Z_{d}=\sqrt{\frac{k}{C_{v}\;\omega }}$$

Here, *C*_*v*_ is the volumetric heat capacity (J m^−3^ K^−1^) of regolith, which is a product of specific heat capacity *c*_*p*_ (J kg^−1^ K^−1^) and bulk density *ρ* (kg m-3), i.e. *C*_*v*_ = *ρ c*_*p*_. Physically, *Z*_*d*_ is the depth, where the diurnal amplitude of the surface heat wave is reduced by a factor of *e* (i.e. 2.718 times). Another important physical property of the regolith is the thermal inertia defined as $I_{T}=\sqrt{kC_{v}}$ (J m^−2^ K^−1^ s^-1/2^). It measures the ‘resistance’ of the substrate’s temperature to change. The higher the thermal inertia the more energy the regolith must absorb in order to increase its temperature by 1 K. The damping depth can also be expressed in terms of thermal inertia as

26 $$Z_{d}=\frac{I_{T}}{C_{v}\sqrt{\omega }}$$

Since both *c*_*p*_ and *k* of the lunar regolith increase with temperature at about the same rate (Hemingway et al. 1973; Vasavada et al. 2012), *Z*_*d*_ has almost no sensitivity to temperature. However, since thermal conductivity strongly depends on air pressure (Presley and Christensen 1997), *Z*_*d*_ varies broadly between Earth, Mars and the Moon primarily as a function of atmospheric pressure. The sensitivity of *Z*_*d*_ to variations in *ω* increases with air pressure and the thermal conductivity of regolith.

The diurnal amplitude of the heat-flux wave (*M*_*s*_) decreases exponentially with regolith depth and planet’s spin rate according to the formula (Ochsner et al. 2007):

27 $$\begin{array}{ll} M\left(z,\omega \right) & =M_{s}\;\exp\left(-z/Z_{d}\right)\\ =M_{s}\;\exp\left(-z\sqrt{C_{v}\;\omega /k}\right) \end{array}$$

where *M*(*z*, *ω*) is the amplitude at depth *z* and rotational frequency *ω*, and *M*_*s*_ is the heat-wave amplitude at the surface. The relative rate of change in *M*(*z*, *ω*) with *ω* is given by the first partial derivative of Eq. (27):

28 $$\frac{\partial M_{z}}{\partial \omega }=-0.5\;z\;\sqrt{C_{v}/\left(\omega k\right)}\;\exp\left(-z\sqrt{C_{v}\;\omega /k}\right)$$

where *M*_*z*_ = *M*(*z*, *ω*)/*M*_*s*_. Equations (27) and (28) suggest that an increasing spin rate has a greater impact of reducing the subterranean diurnal temperature amplitude near the surface than it does at depth. This implies that the *annual mean* temperature of the subsurface is not impacted by *ω*. A planet spin rate also affects the time lag between heat flux maxima at the surface and at depth *z*. This lag decreases in proportion to $1/\sqrt{\omega }$ (Ochsner et al. 2007), which means that a faster rate of rotation brings the subsurface heat wave closer in phase to the surface heat wave.

Equation (25) through (28) collectively suggest that a faster rotational speed causes less heat to be conducted into the ground under steady-state conditions. This is due to a reduction of the vertical temperature gradient at the surface, which is a key factor controlling the magnitude of instantaneous ground heat fluxes (Eq. 21). The vertical gradient ∂*T*/∂*z* reaches maximum for a particular illumination when the incident solar flux changes slow enough to stay in equilibrium with the outgoing infrared flux. Increasing the rotational speed causes incident solar radiation to vary faster than the rate of ∂*T*/∂*z* formation set by the regolith thermal inertia. As a result, the temperature gradient begins to deviate from its potential strength. The faster the rotation, the greater the deviation of ∂*T*/∂*z* would be for a given ground thermal inertia. Thus, increasing a planet’s angular velocity *decreases* the average ground heat flux via reduction of instantaneous heat fluxes. This causes the ratio in Eq. (24), i.e. the solar flux fraction stored into the ground to remain conservative across planet spin rates. The Law of Energy Conservation dictates that a change in rotational speed may only affect the magnitude of the diurnal temperature amplitude at the surface but not the diurnal mean, i.e. rotation solely acts to redistribute the total available energy between daytime and nighttime hemispheres through the planet’s thermal inertia. As the rotation frequency increases beyond a certain threshold, the surface temperature amplitude begins to shrink, thus flattening the diurnal heat wave without affecting the diurnal mean. This frequency threshold depends on the absolute magnitude of the surface thermal inertia, i.e. the greater the inertia the slower the rotational speed, at which the diurnal temperature amplitude becomes affected. Since regolith-covered ASCOs such as the Moon are expected to have a very small thermal inertia due to a low thermal conductivity of the regolith in vacuum, it takes a rather fast axial rotation to noticeably impact the diurnal temperature amplitude at the surface. This analysis suggests that *ω* cannot affect *η*_*e*_ and the average surface temperature of a planet. The heat storage fraction can only be altered by a significant change in the apparent thermal conductivity of the regolith, which requires the introduction of a qualitatively different environment such as adding atmospheric pressure to the surface.

### Magnitude and components of Earth’s atmospheric thermal effect

The above discussion leads to the conclusion that Earth’s *total* ATE must be evaluated with respect to Earth’s hypothetical *airless* self. The thermal environment of the Moon offers a perfect natural airless equivalent of our Planet. Therefore, using either *T*_na_ = 197.1 K calculated from Eq. (16) or *T*_*moon*_ = 197.3 K derived from the TWO thermo-physical model, we obtain:

$$\mathrm{ATE}=T_{s}-T_{na}=287.6-197.1=90.5K$$

and

$$\mathrm{ATE}=T_{s}-T_{\mathrm{moon}}=287.6-197.3=90.3K$$

Accepting Eq. (16) as the proper analytic model for calculating the average surface temperature of airless spherical bodies allows us to produce an alternative estimate of *T*_na_ using Earth’s present surface albedo of 0.122 inferred from satellite observations (Stephens et al. 2012). The result is *T*_na_ = 197.6 K, which translates into ATE = 287.6–197.6 = 90.0 K. This is only 0.3K–0.5 K lower than the above ATE estimates based on the Moon albedo of 0.13. In order to claim robustness of our ATE estimate, however, we must evaluate the uncertainty of *T*_na_ associated with a plausible range of Earth albedos in the absence of atmosphere. There is currently no rigorous quantitative method available for calculating the albedo of a hypothetical airless Earth; hence, one must use physical reasoning to obtain a proper value range. Two conditions need be met when utilizing observational data from airless bodies in the Solar System to make a logical inference about Earth’s albedo without an atmosphere:

1. One must only consider the regolith albedos of *ice-free* airless bodies. This is so, because the solar heating at Earth’s orbit is strong enough to quickly evaporate any exposed water ice on an airless surface. Ices of other gases such as CO_2_, CH_4_ and nitrogen also cannot form under a solar irradiance of 1,361 W m^−2^. It is for this reason that significant amounts of water ice are only found on the airless Moon in permanently shadowed craters near the lunar poles (Colaprete et al. 2010; Spudis et al. 2010). Hence, this condition excludes from consideration the high albedos of airless icy bodies such as Saturn’s satellites Rhea, Dione, Tethys, Mimas, and Enceladus as well as Jupiter’s moon Europa;
2. One should consider bolometric Bond albedos rather than geometric albedos in global energy-budget calculations. This is because Bond albedos are spherical, while geometric albedos are directional. Studies oftentimes only report geometric albedos of celestial objects, since these are directly measurable, while Bond albedos must be calculated and require knowledge of the hemispheric phase integral. Airless bodies usually have Bond albedos that are lower than their geometric albedos.

Available surface reflectance data from the Solar System suggest that bolometric Bond albedos of ice-free regolith-covered airless bodies typically range from 0.068 to 0.16 (Mallama et al. 2002; Shestopalov and Golubeva 2011). Some small-sized asteroids mostly composed of rocks have lower Bond albedos than 0.068, but such values are not typical for larger regolith-covered bodies. Employing the above albedo limits with Eq. (16) yields a global average temperature for a hypothetical airless Earth 195.4 K ≤ *T*_na_ ≤ 200.6 K. This translates into an ATE between 87.0 K and 92.2 K. Thus, one can formally quote Earth’s ATE as 89.6 ± 2.6 K, although we consider ATE = 90.5 K to be our best estimate. Therefore, the thermal effect of our atmosphere is 2.7 to 5 times stronger than currently assumed based on Eq. (3). According to our analysis, Earth’s ATE varies spatially from 86 K at the Equator to about 148 K at the Poles.

In order to assess the contribution of Earth’s present surface heat storage to the planet’s global temperature, we set *η*_*e*_ = 0.075 and *α*_*e*_ = 0.294 in Eq. (16). The result is a new reference temperature *T*_na2_ = 213.0 K that is 15.7 K higher than the Moon’s present airless temperature (our true reference). This thermal enhancement is caused by a 7.7-fold increase of *η*_*e*_ above the corresponding lunar value. The larger heat storage fraction reflects the presence of an atmosphere and is a consequence of a much higher surface thermal conductivity on Earth due to air pressure. Note that increasing the albedo from 0.131 to 0.294 only partially offsets the enhancement effect of a larger *η*_*e*_ on global temperature. Thus, the daytime storage of heat by landmasses and oceans on Earth significantly contributes to our planet’s ATE by raising the average nighttime temperatures. This implies that Earth’s atmospheric effect has a sizable thermodynamic component that is independent of the greenhouse infrared back radiation. In other words, ATE includes more than just the radiative effect of greenhouse gases (GE), i.e. ATE = GE + TE, where TE is a Temperature Enhancement caused by thermodynamic (pressure-controlled) processes. The thermal effect of radiatively active gases is then obtained as a residual, i.e. GE = ATE – TE.

We must make an important clarification regarding the above decomposition of ATE. The subterranean heat storage (considered in our model) boosts the global surface temperature by effectively ‘transporting’ a fraction of the daytime absorbed solar flux to the night side of the planet via axial rotation. In the presence of atmosphere, however, the air- and oceanic currents (not considered in our airless model) will foster additional lateral transfer of heat that further increases the planet’s average temperature. This energy transport via fluid motion includes advective and radiative components that are difficult to separate through a simple analysis. Hence, the thermodynamic portion of Earth’s ATE is likely greater than 15.7 K in reality. However, an accurate assessment of the TE magnitude is a non-trivial task and entails the use of coupled atmosphere–ocean global circulation models, which is beyond the scope of this study. Therefore, the above TE estimate (~16 K) should merely be viewed as an indication that Earth’s ATE has indeed a sizable thermodynamic component requiring further investigation. Indirect support for the existence of TE is also provided by the fact that the observed 158 W m^−2^ global atmospheric absorption of outgoing long-wave radiation (Stephens et al. 2012; Wild et al. 2013) cannot fully explain the hereto deduced ~90 K total ATE. The TE component inferred from our model analysis offers a new premise to the Greenhouse theory, which currently attributes 100% of Earth’s ATE to an infrared heat trapping by greenhouse gases (e.g. Hansen et al. 1981; Peixoto and Oort 1992; Schmidt et al. 2010; Lacis et al. 2013).

We surmise that the radiative portion of ATE controlled by greenhouse gases might be larger than 33 K in reality. Such a conjecture is supported by a recent simulation study of Russell et al. (2013), which found using a streamlined 3-D coupled atmosphere–ocean model that Earth's global temperature would drop 44.4 C (to −30 C) if the atmospheric CO_2_ concentration were reduced to 1/8 of its 1950 level. However, these authors did not examine the change in modeled global temperature under an atmosphere completely devoid of greenhouse gases. Had they done so, the outcome would probably have been a greater surface cooling than 44 C. These simulation results (if correct) when combined with our findings indicate that the long-term impact of increasing greenhouse-gas emissions on climate might be stronger than currently projected. Indeed, recent paleoclimate studies comparing proxy-derived temperatures and CO_2_ concentrations for the mid-Pliocene to simulations by fully coupled models (Lunt et al. 2010; Haywood et al. 2013) found that the Earth System Sensitivity (ESS) (defined as the equilibrium global surface temperature response to a sustained doubling of atmospheric CO_2_ concentration including *all* feedbacks) might be 1.5 times higher than the conventional climate sensitivity to CO_2_ and the water-vapor feedback simulated by climate models. Paleoclimate studies have also suggested that ESS might depend on the state of the climate system (e.g. Caballero and Huber 2013).

## Conclusions

The observed global energy balance of celestial bodies is often used in conjunction with a simple form of the SB radiation law (Eq. 3) to calculate equilibrium effective radiating temperatures (*T*_e_) that find a broad application in today’s climate and planetary sciences. For more than 35 years, these calculated temperatures have been utilized to compare the thermal regimes of airless bodies, to quantify the strength of atmospheric greenhouse effects, and to assess the potential habitability of extrasolar planets. Thus, Earth’s effective radiating temperature of ~255 K, which includes the albedo effect of clouds, is the basis for the popular 33 K estimate of the total background atmospheric warming a.k.a. Natural Greenhouse Effect. Although *T*_e_ is derived from a well-known physical law, a close examination of the meaning of this temperature in the context of planetary energetics and spherical geometry reveals two critical caveats. First, a global emission temperature computed from Eq. (3) using the planet’s actual Bond albedo that includes the radiative effects of greenhouse gases and air molecules, cannot serve as a proper reference in quantifying the thermal effect of a planetary atmosphere. This is because a reference temperature is expected in this case to describe a thermal state in the *absence* of greenhouse gases or an atmosphere. However, Earth’s 33 K GE estimate is based on a *T*_e_ value that violates such a condition. Zeng (2010) recognized this and proposed using an average land-ocean *surface* albedo in Eq. (3) instead of Earth’s Bond albedo as a solution. He essentially argued that the appropriate reference temperature for calculating Earth’s ATE is that of an airless Earth, which we concur with. Employing a 0.14 surface albedo in Eq. (3) Zeng arrived at GE ≈ 20 K. However, this author did not address another more fundamental problem of Eq. (3) related to Hölder’s inequality between integrals. The non-linearity of the SB radiation law coupled with a strong latitudinal variation of the absorbed solar flux across the surface of a sphere creates a mathematical condition that precludes *in principle* a correct calculation of the true global surface temperature from a spatially integrated radiative flux. In other words, due to Hölder’s inequality, one always finds *T*_e_ ≫ *T*_na_. Leconte et al. (2013) acknowledged this phenomenon, but the actual magnitude of the inequality and its theoretical implications have not been fully analyzed prior to our study. We showed that the actual mean surface temperature of the Moon (197.3 K) is about 73 K cooler than the Moon’s effective radiating temperature *T*_e_ ≈ 270 K computed from Eq. (3) using the same albedo. This large discrepancy is due to the fact that Eq. (3) essentially yields a disk-average temperature instead of a spherical temperature mean. Most studies treat globally averaged radiative fluxes and their corresponding effective radiating temperatures as physically interchangeable quantities (e.g. Lacis et al. 2013). However, according to Hölder’s inequality (4b), this is conceptually incorrect. While the average outgoing LW flux of a planet is an observable physical parameter, *T*_e_ derived from it using Eq. (3) is a mathematical abstraction with no physical analogue at, below or above the surface. Thus, planetary effective emission temperatures are not compatible with measured physical temperatures regardless of the albedo they are based on. In other words, *T*_e_ is a non-physical quantity with respect to a sphere. Consequently, comparing Earth’s observed global mean surface air temperature (287.6 K) to any *T*_e_ is bound to produce numerically and theoretically misleading results. The conceptual distinction between *T*_e_ on one hand and *T*_na_ or *T*_s_ on the other arises from the mathematical understanding that mean planetary temperatures cannot in principle be inferred from globally averaged radiative fluxes. In this regard, our analysis demonstrates that evaluating the strength of a planet’s ATE strictly requires the use of physical surface temperatures.

To properly account for Hölder’s inequality, we derived a new expression for the mean surface temperature of airless bodies (Eq. 10 and 11a) based on analytic integration of the SB law over a sphere with explicit consideration of the effects of regolith heat storage and cosmic background radiation on nighttime temperatures. The new model was successfully verified against independent temperature data for the Moon provided by the Diviner Lunar Radiometer Experiment. Verification results suggested a small adjustment to the nighttime integration. The final equations (14) and (16) provide a substantially improved method for quantifying the average temperatures of airless bodies compared to the current SB formula (3). An error analysis employing the new model revealed that Eq. (3) overestimates the global physical temperature of airless bodies by about 37% with the absolute error increasing proportionally to the 4th root of the TOA stellar irradiance. This large bias follows from functional differences between equations (3) and (16). Based on these results, we propose equations (14) and (16) as a new analytic standard for calculating the average surface temperatures of airless bodies.

We presented evidence that the Moon is a perfect airless grey-body equivalent of Earth. A key element of this evidence is that the regolith heat storage fraction *η*_*e*_, which has a critical impact on the global temperature (Eq. 16), strongly depends on atmospheric pressure through the surface thermal conductivity. We showed that air pressure significantly boosts the heat storage capacity of Earth compared to the lunar environment and significantly contributes to the overall thermal effect of our atmosphere. The presence of such a large thermodynamic component (TE) implies that, when it comes to assessing the total magnitude of ATE, an Earth *with* an atmosphere devoid of greenhouse gases is not physically equivalent to an Earth *without* an atmosphere. Hence, the *overall* thermal effect of a planetary atmosphere should be evaluated with respect to the mean surface temperature of an equivalent *airless* body calculated from Eq. (14) or (16). Combining Earth’s observed global surface temperature with results from the new analytic model reveals that the total thermal effect of our atmosphere is about 90 K or 2.7 to 5 times stronger than currently assumed. At least 17% (15.7 K) of this ATE is due to thermodynamic factors that are independent of the atmospheric infrared back radiation. The non-radiative portion of Earth’s ATE is likely greater than 15.7 K in reality due to horizontal heat transports by oceanic and atmospheric currents not considered in our model. The hereto identified thermodynamic component of ATE creates a new premise for the Greenhouse theory, which currently attributes 100% of the background atmospheric warming to a long-wave radiation trapping by greenhouse gases. Finally, our analysis suggests that the exact contribution of heat-absorbing gases to Earth’s atmospheric effect will remain unknown until the non-radiative component of ATE is fully quantified. Therefore, further fundamental research is needed in atmospheric radiative transfer and 3-D tropospheric thermodynamics to better constrain the functional elements of Earth’s atmospheric thermal effect.

## Authors’ information

The authors have PhD Degrees in physical sciences and work as environmental consultants for a non-profit organization in Utah, USA.

## Authors’ original submitted files for images

Below are the links to the authors’ original submitted files for images. Authors’ original file for figure 1 Authors’ original file for figure 2 Authors’ original file for figure 3 Authors’ original file for figure 4 Authors’ original file for figure 5 Authors’ original file for figure 6 Authors’ original file for figure 7 Authors’ original file for figure 8 Authors’ original file for figure 9 Authors’ original file for figure 10
