# Erratum to: On the average temperature of airless spherical bodies and the magnitude of Earth’s atmospheric thermal effect

**DOI:** 10.1186/s40064-016-3755-3

**Published:** 2016-12-09

**Authors:** Ned Nikolov, Karl Zeller

**Affiliations:** Tso Consulting, 843 E Three Fountains Suite 260, Salt Lake City, UT 84107 USA

## Erratum to: SpringerPlus (2014) 3:723 DOI 10.1186/2193-1801-3-723

As authors of this article (Volokin and ReLlez 2014) we would like to clarify that our real names are Ned Nikolov and Karl Zeller. We created the pseudonyms Den Volokin and Lark ReLlez by spelling our names backward. Ned Nikolov is a physical scientist with the USDA Forest Service; he had been instructed by his employer not to engage in climate research during government work hours, nor to reveal his government affiliation when presenting results from his climate studies. Karl Zeller is a retired USDA Forest Service research scientist with no restrictions. Ned Nikolov worked on this manuscript outside of his assigned official work duty hours. Because of the controversial subject matter and the novel findings previously associated with Nikolov and Zeller, we felt that the use of pseudonyms was necessary to guarantee a double-blind peer review of our manuscript and to assure a fair and unbiased assessment. We are sorry for any inconvenience this may have caused the Editorial Board and the readership of *SpringerPlus*.

## References

[CR1] Volokin D, ReLlez L (2014). On the average temperature of airless spherical bodies and the magnitude of Earth’s atmospheric thermal effect. SpringerPlus.

