# Supplementary figures and images for: On the average temperature of airless spherical bodies and the magnitude of Earth’s atmospheric thermal effect
Source: Springerplus. 2014 Dec 10;3(1):723. doi: 10.1186/2193-1801-3-723 (PMC4447774; doi:10.1186/2193-1801-3-723)

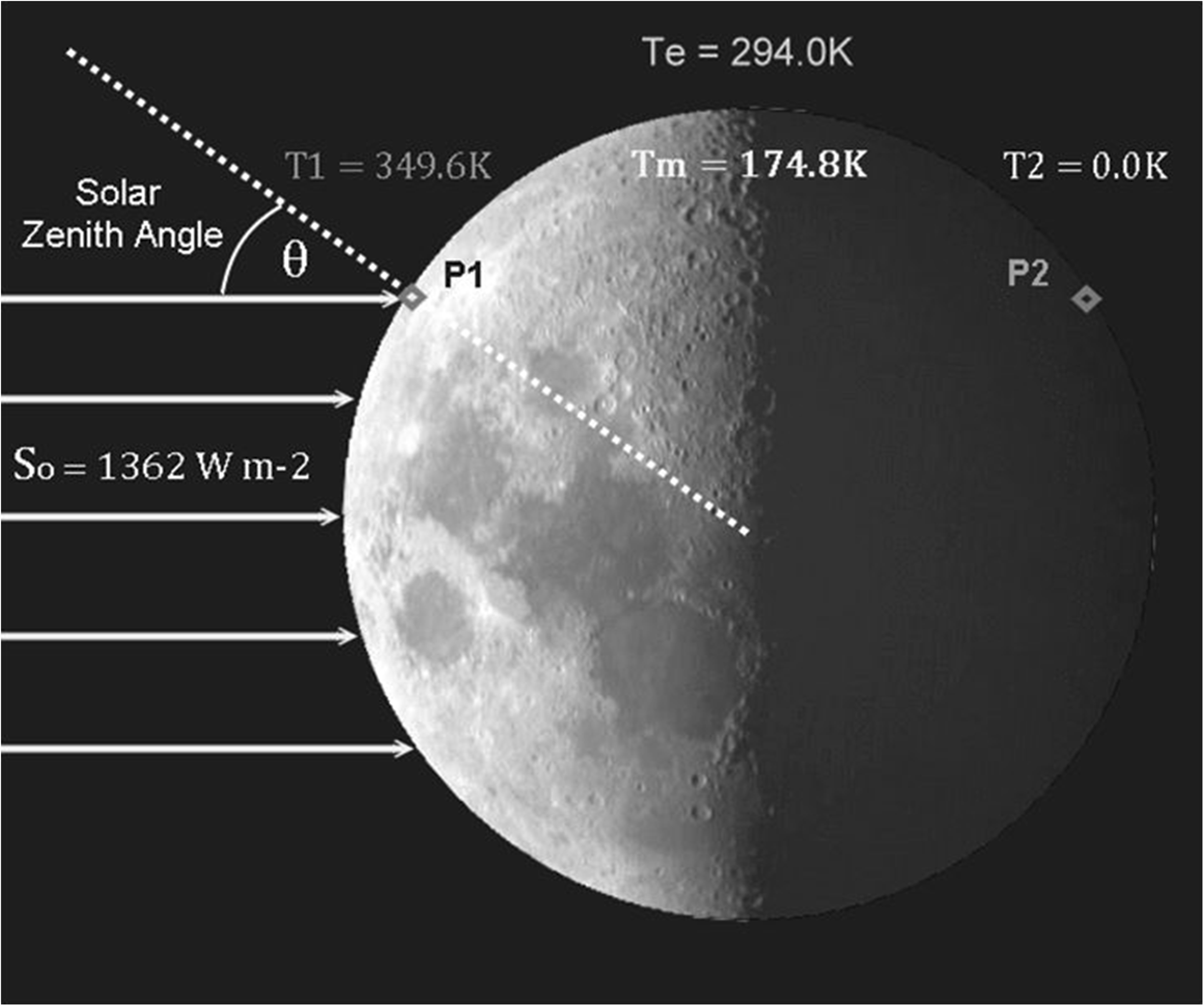

Supplement: Supplementary file 1 — Authors’ original file for figure 1 [file 40064_2014_1586_MOESM1_ESM.tif]

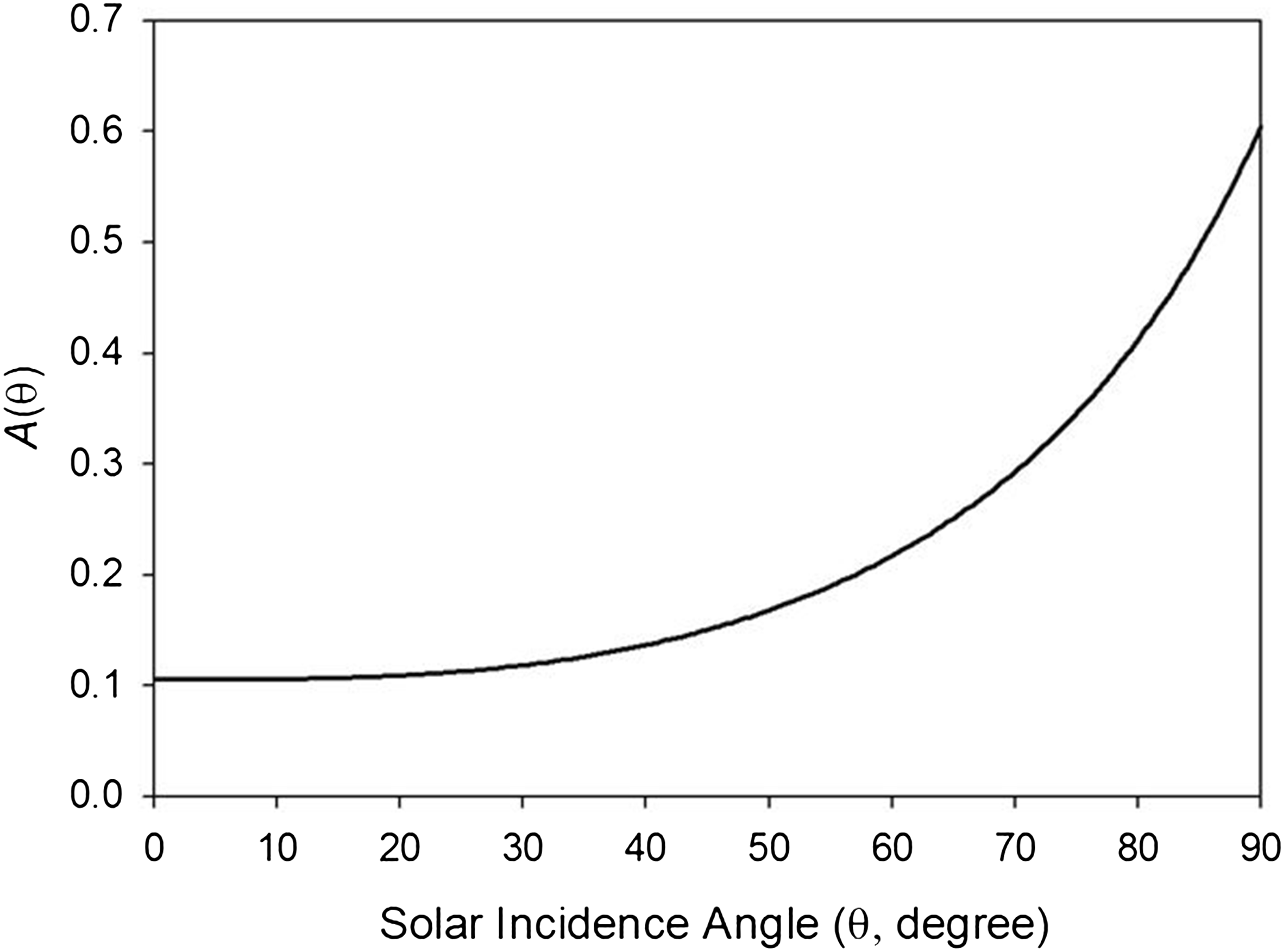

Supplement: Supplementary file 2 — Authors’ original file for figure 2 [file 40064_2014_1586_MOESM2_ESM.tif]

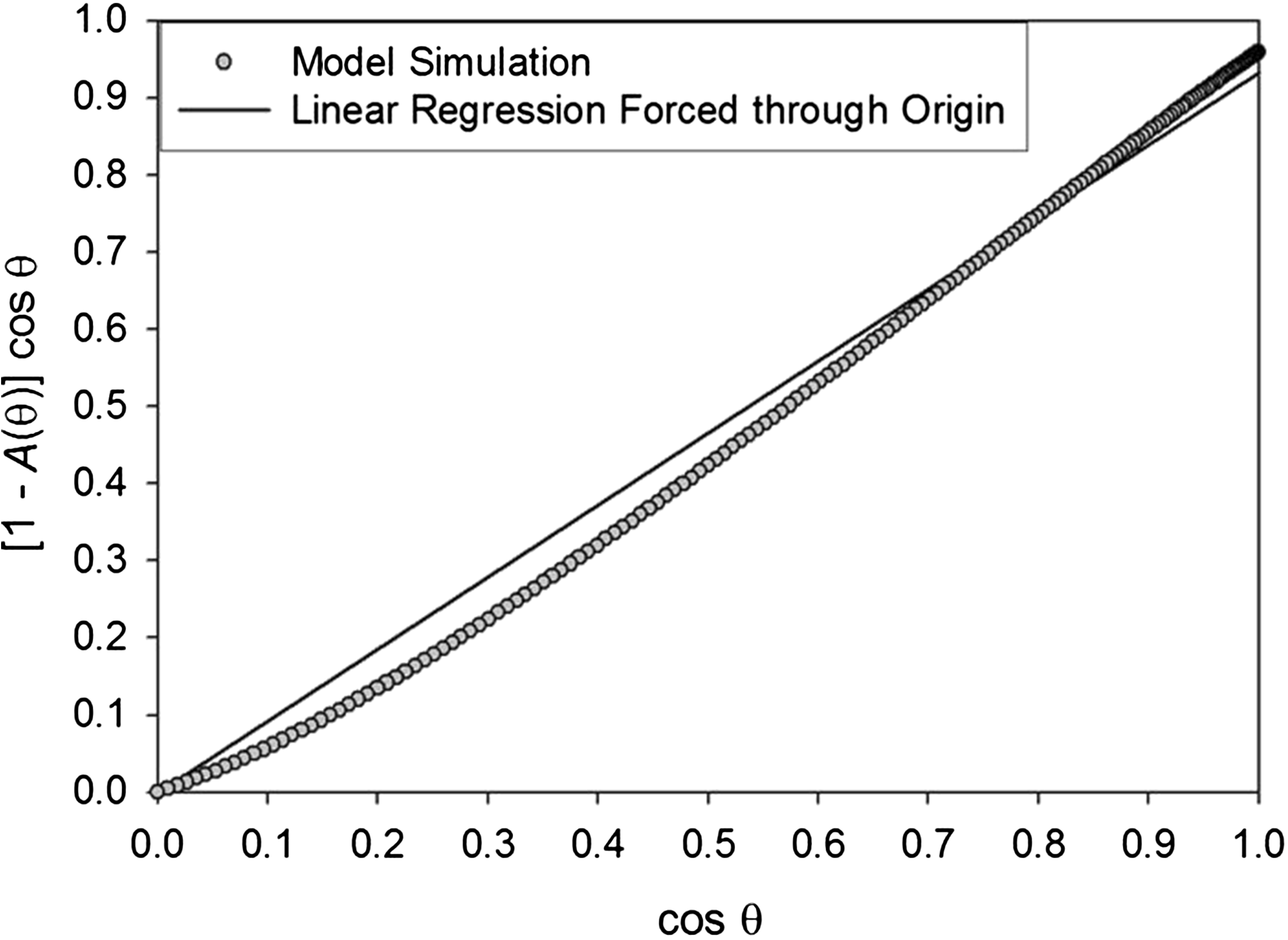

Supplement: Supplementary file 3 — Authors’ original file for figure 3 [file 40064_2014_1586_MOESM3_ESM.tif]

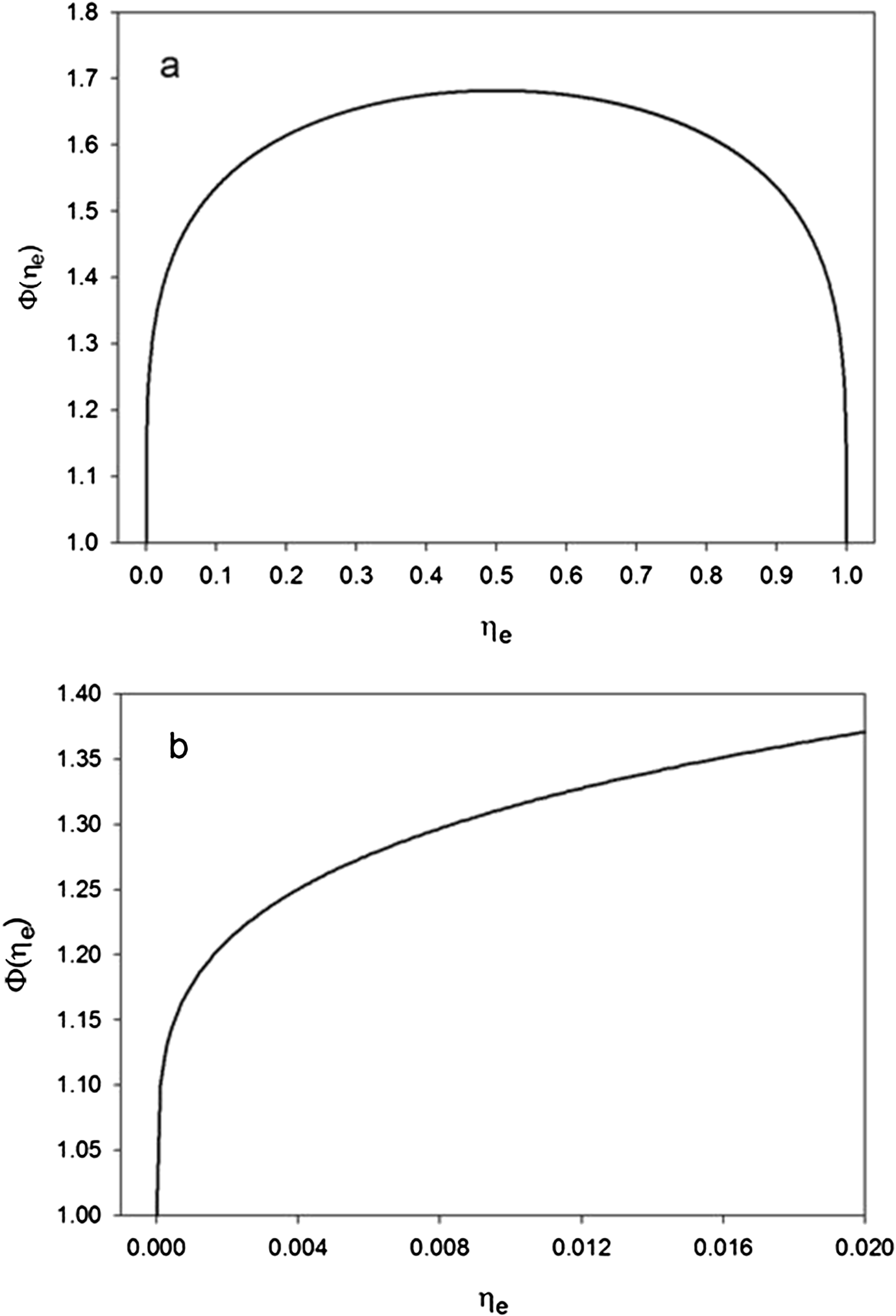

Supplement: Supplementary file 4 — Authors’ original file for figure 4 [file 40064_2014_1586_MOESM4_ESM.tif]

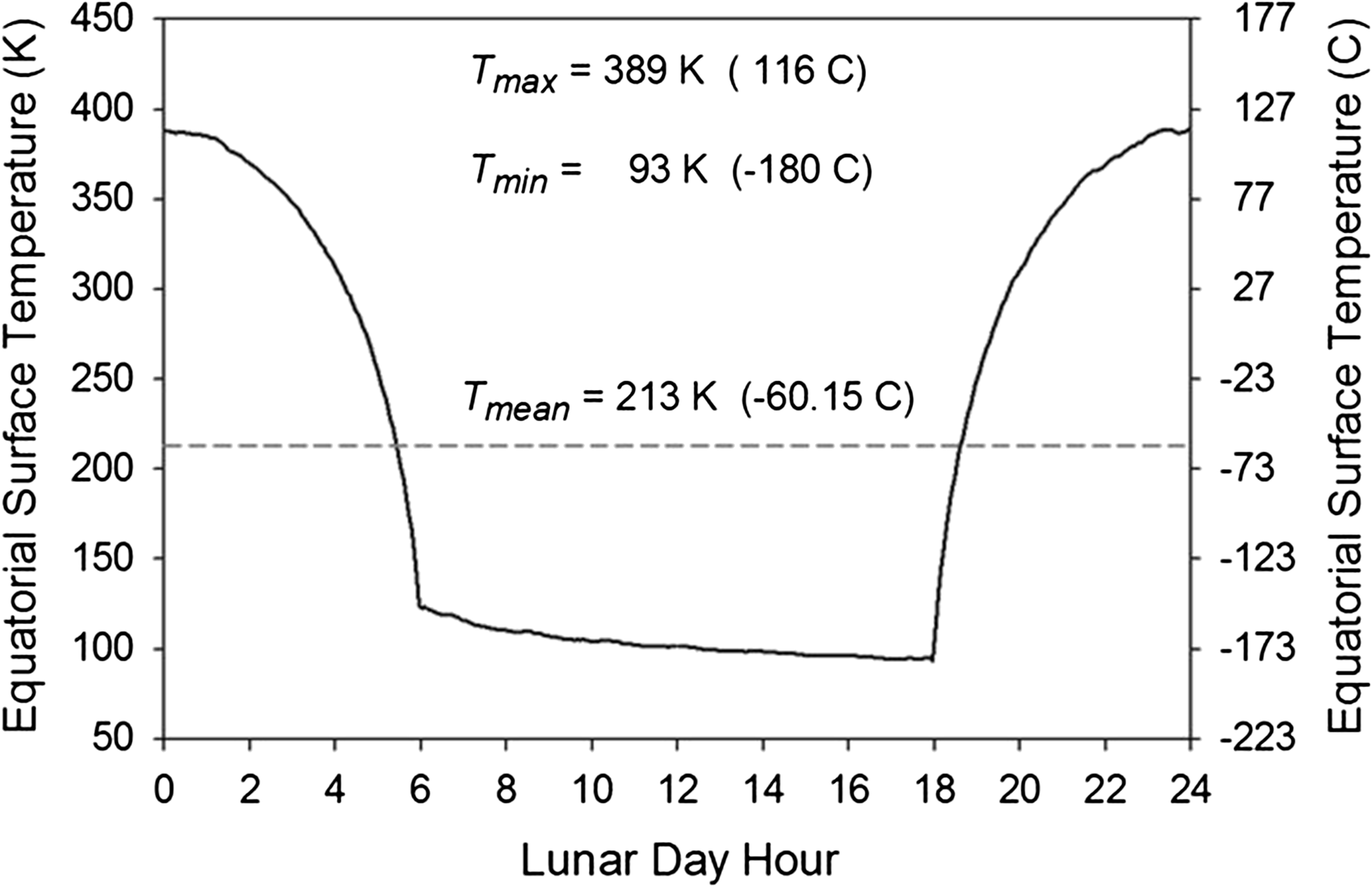

Supplement: Supplementary file 5 — Authors’ original file for figure 5 [file 40064_2014_1586_MOESM5_ESM.tif]

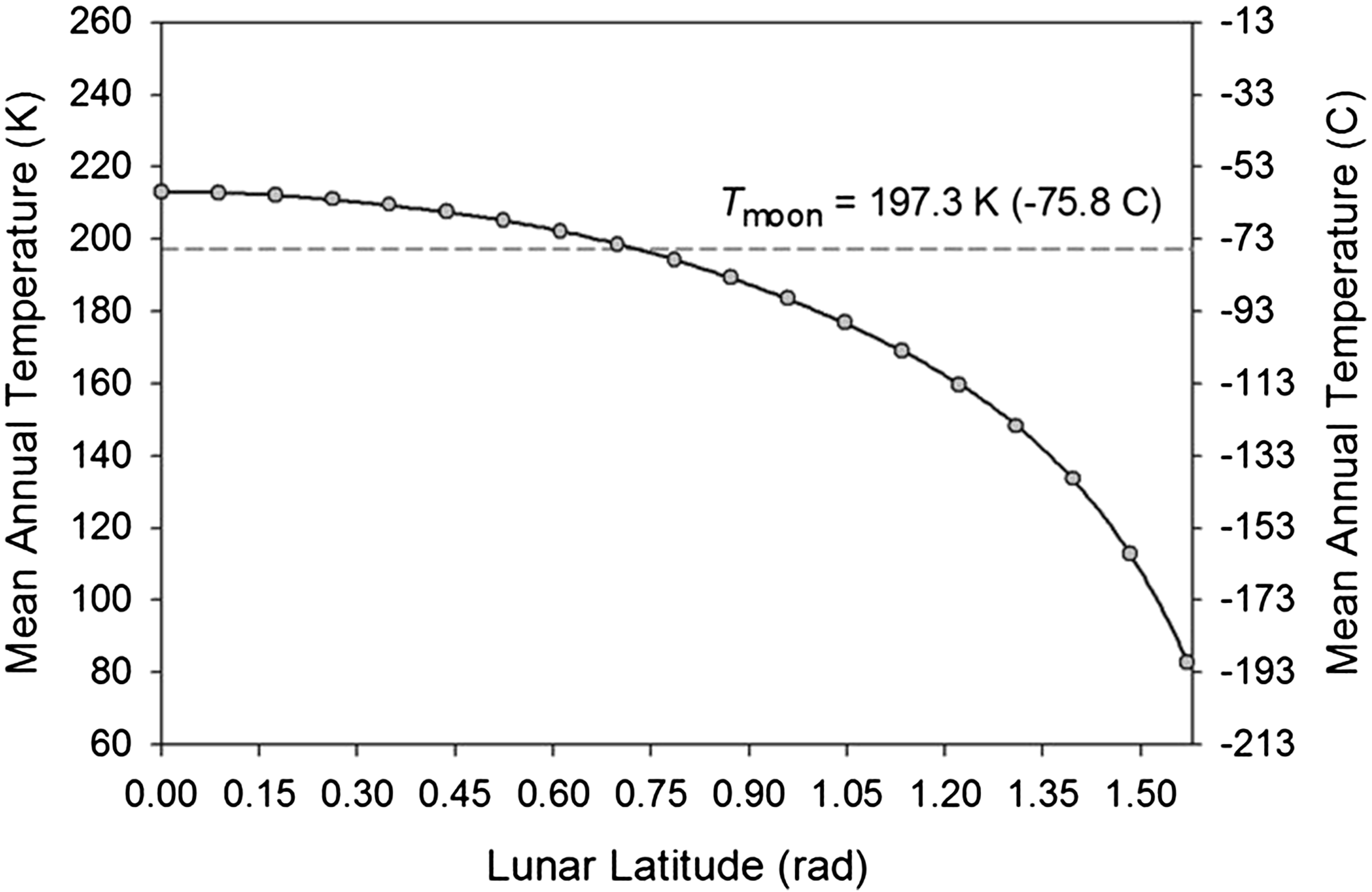

Supplement: Supplementary file 6 — Authors’ original file for figure 6 [file 40064_2014_1586_MOESM6_ESM.tif]

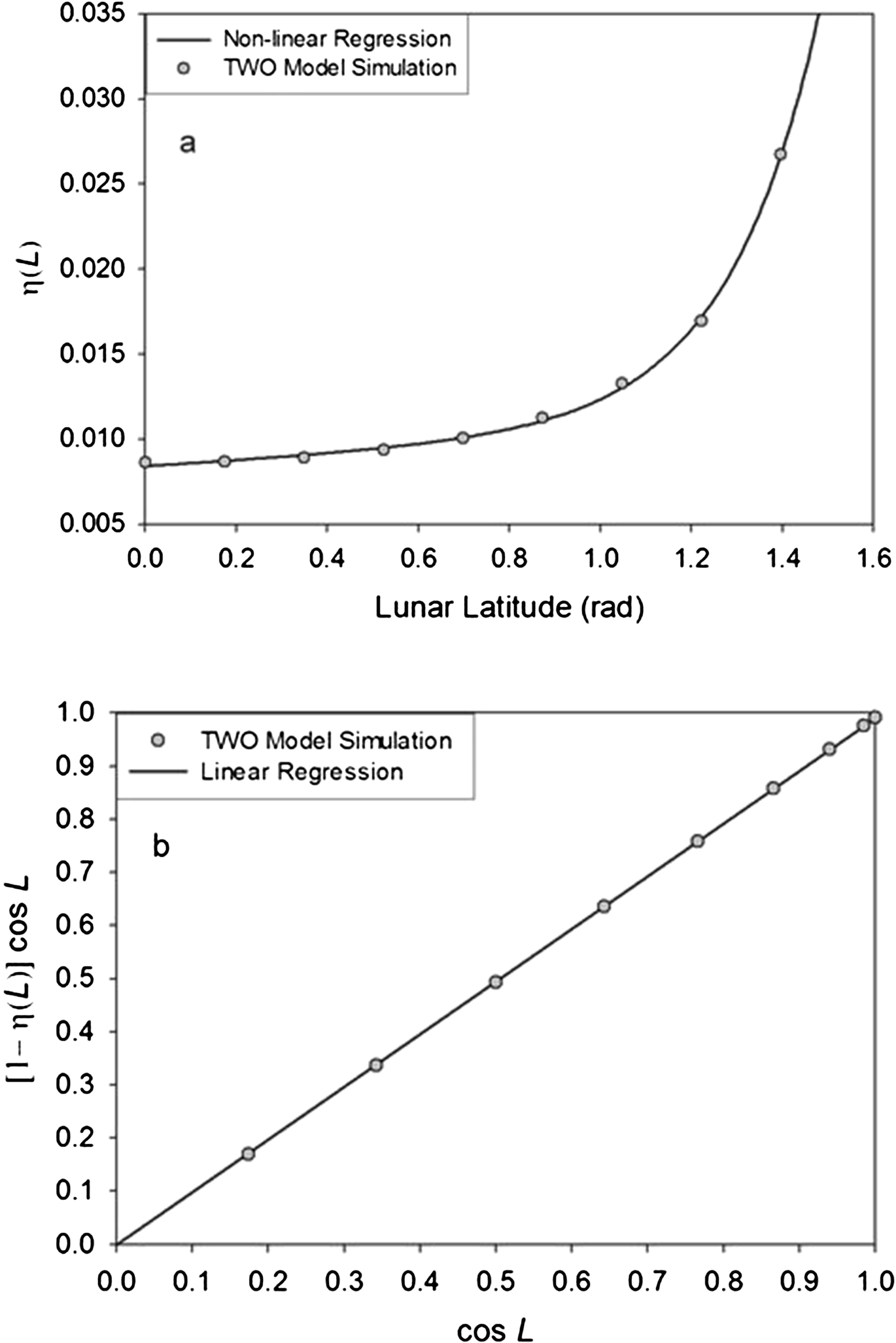

Supplement: Supplementary file 7 — Authors’ original file for figure 7 [file 40064_2014_1586_MOESM7_ESM.tif]

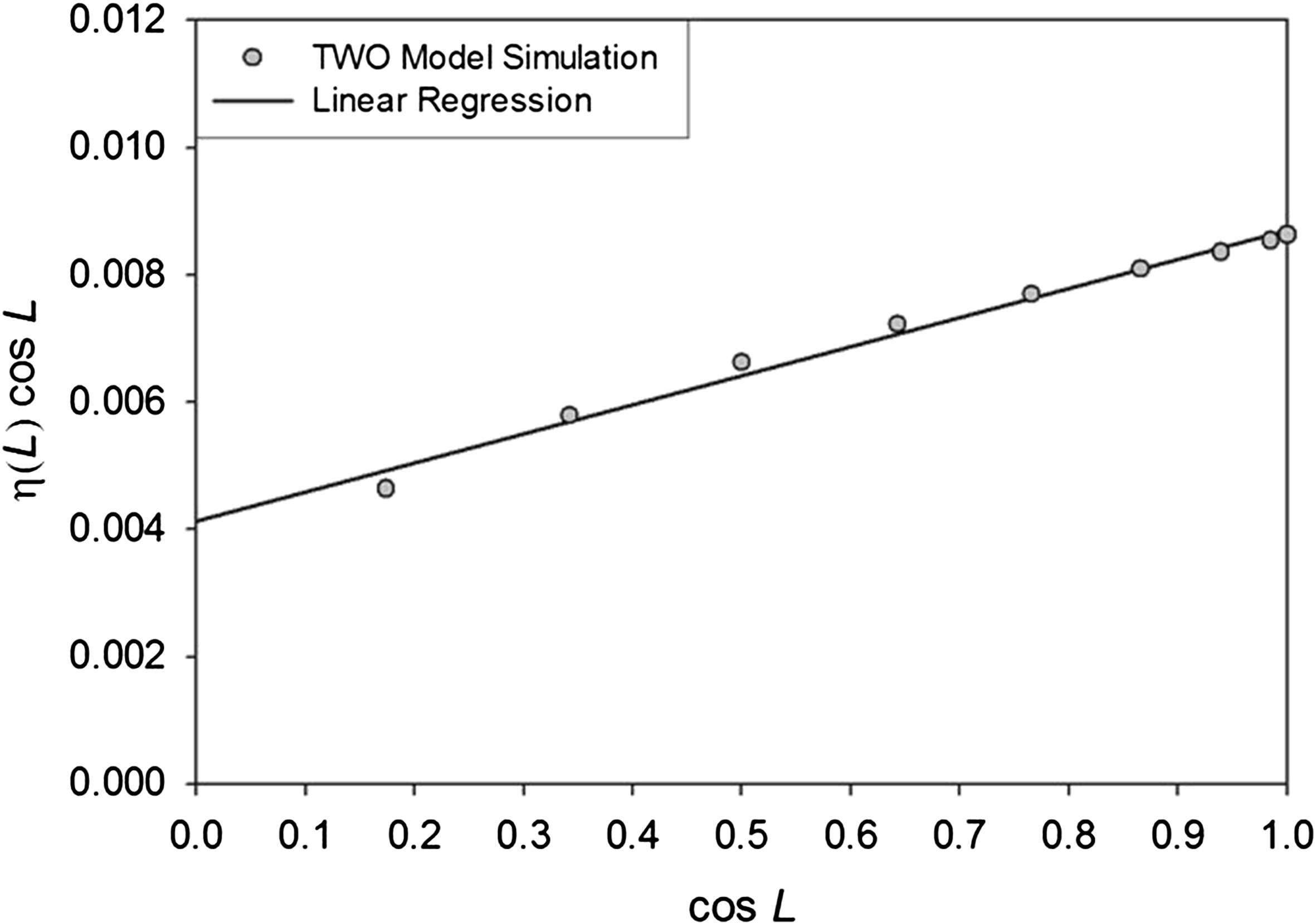

Supplement: Supplementary file 8 — Authors’ original file for figure 8 [file 40064_2014_1586_MOESM8_ESM.tif]
